# Comprehensive multiomic analysis of extracellular vesicles from *Mycoplasma bovis*-infected bovine mammary epithelial cells identifies proteins and miRNAs that induce inflammatory responses in macrophages

**DOI:** 10.1186/s13567-025-01626-5

**Published:** 2025-09-25

**Authors:** Yiming Wu, Xiaotan Yuan, Jiating Ma, Lihua Xu, Min Li, Gang Zhao, Yujiong Wang

**Affiliations:** 1 Key Laboratory of the Ministry of Education for Conservation and Utilization of Special Biological Resources in the Western Region, Ningxia University, Yinchuan, 750021 China; 2 College of Life Science, Ningxia University, Yinchuan, 750021 Ningxia China; 3 College of Animal Science, Ningxia University, Yinchuan, 750021 Ningxia China

**Keywords:** *M. bovis*, extracellular vesicles, inflammatory response, proteomics, transcriptomics

## Abstract

*Mycoplasma bovis* can lead to a decline in milk quality and yield, thereby causing significant economic losses worldwide. Extracellular vesicles (EVs) are crucial for triggering immune cell responses to infection. This study aimed to demonstrate the immunomodulatory effects of EVs released by bovine mammary epithelial cells (MAC-T cells) infected with *M. bovis* on bovine macrophages (BoMacs). After EVs were extracted from *M. bovis*-infected MAC-T cells (*M. bovis* NX2-EVs) as well as from uninfected MAC-T cells (Ctrl-EVs), they were incubated with BoMacs to assess their potential to induce cytokine expression. The results showed that *M. bovis* NX2-EV-treated BoMacs exhibited significantly increased expression of TNF-α, IL-1β, and IL-6. Additionally, the differentially expressed genes mainly involved the TNF, NF-kappa B and IL-17 signalling pathways, with endocytosis and megalocytosis recognized as the main pathways through which BoMacs can take up EVs. Furthermore, mass spectrometry and RNA-seq were used to determine the protein and miRNA expression profiles of Ctrl-EVs and *M. bovis* NX2-EVs. Overall, 27 And 86 proteins were significantly downregulated and upregulated, respectively, in *M. bovis* NX2-EVs compared with those in Ctrl-EVs. Similarly, a total of 9 miRNAs were upregulated, while 2 miRNAs were downregulated in *M. bovis* NX2-EVs. Finally, JCHAIN, MAPRE1, miR-1307, and miR-149-5p were identified as differentially expressed proteins and miRNAs in *M. bovis* NX2-EVs, thus highlighting their involvement in cellular immune regulation and related diseases. These results reveal the mechanism of host resistance to *M. bovis* infection and provide new insights for exploring the pathogenic mechanism of *M. bovis.*

**Supplementary Information:**

The online version contains supplementary material available at 10.1186/s13567-025-01626-5.

## Introduction

*Mycoplasma bovis* is currently recognized as a highly significant and frequently encountered *Mycoplasma* species associated with bovine diseases [1, 2], such as pneumonia [3], mastitis [4] and arthritis [5]. Since its discovery in 1961, it has become widely prevalent in cattle-farming countries around the world [6], and as a result, *M. bovis*-induced mastitis is now a global issue that leads to substantial reductions in milk production as well as significant economic losses [7]. Numerous studies have shown that the pathogenesis of *M. bovis* infection involves immune evasion and the modulation of host immune responses that subsequently trigger an inflammatory response [8, 9]. In fact, the membrane lipoproteins of this *Mycoplasma* species are believed to play an essential role in inducing such proinflammatory responses [10]. Additionally, certain *M. bovis* metabolites can induce host inflammatory responses, leading to cellular damage and intensified inflammatory reactions [11–14]. However, no studies have explored the inflammatory response induced by extracellular vesicles (EVs) derived from *M. bovis*-infected cells.

EVs are lipid bilayer-enclosed nanoparticles secreted by cells and are typically categorized as apoptotic bodies, microvesicles or exosomes, depending on their size [15]. Previous research has suggested that EVs are involved in various inflammatory diseases mainly through the delivery of proteins or miRNAs [16, 17]. Furthermore, EVs derived from pathogen-infected host cells play a regulatory role in the immune response by participating in antigen presentation, thereby activating immune cells (e.g., macrophages and natural killer (NK) cells) to stimulate the release of inflammatory mediators [18, 19]. Moreover, miRNAs in EVs serve as a critical mechanism for exosome-mediated inflammatory responses. For instance, in inflammatory conditions, such as inflammatory bowel disease (IBD) [20] and acute kidney injury (AKI) [21], bone marrow mesenchymal stem cell-derived exosomes have shown therapeutic potential. Specifically, EVs that carry miR-539-5P have been shown to alleviate IBD through the inhibition of cellular pyroptosis [22], whereas exosomes containing miRNA-19b-3p and released by lipopolysaccharide (LPS)-stimulated renal tubular epithelial cells contribute to renal injury by promoting M1-type macrophage polarization [23]. The role of miRNAs in exosomes derived from *Mycoplasma*-infected host cells in resistance to *Mycoplasma* infection has been specifically investigated. More interestingly, exosomal miRNAs derived from *Mycoplasma gallisepticum* (MG)-infected CP-II cells play important roles in regulating the production of inflammatory cytokines in DF-1 cells [24]. Similarly, exosomal miR-181a-5p from MG-infected CP-II cells promotes the expression of proinflammatory cytokines by activating the TLR2-mediated MyD88/NF-KB signalling pathway in receptor DF-1 cells, thereby preventing MG-HS infection [25]. These results underscore the significant role of EV miRNAs in modulating inflammatory responses. However, the effects of those from *M. bovis*-infected cells on the regulation of other host cells remain unclear.

Bovine mammary epithelial cells (MAC-T cells), derived from bovine mammary tissues, are typically selected for mastitis-related studies [26]. The protein and miRNA profiles in EVs derived from MAC-T cells have been shown to be consistent with those derived from milk [27]. Thus, MAC-T cells provide a valuable model for research on bovine mastitis. Additionally, BoMacs, derived from bovine macrophages, which are involved in pathogen phagocytosis and clearance, antigen processing and presentation, as well as the release of various cytokines, are essential for both innate and adaptive immunity. In fact, they serve as the primary line of defence after host infection with pathogens [28]. In this study, the effects of EVs derived from *M. bovis*-infected MAC-T cells (designated *M. bovis* NX2-EVs) on the inflammatory response of BoMacs, mouse bone marrow-derived macrophages (BMDMs), and bovine monocyte-derived macrophages were examined. The findings suggest that *M. bovis*-infected MAC-T cells can stimulate inflammatory responses in BoMacs, BMDMs and bovine monocyte-derived macrophages through the release of EVs. Moreover, the proteins and miRNAs in EVs involved in promoting the inflammatory response were further identified. Understanding the cargo of these EVs is expected to offer new insights into their molecular mechanisms of action.

## Materials and methods

### Strain culture

A milk sample, which was collected from an infected beef cow in the Ningxia Hui Autonomous Region, China, was used as the source material for isolating *Mycoplasma bovis* strain NX2 (GenBank: CP171654.1). Following its characterization by our laboratory, the strain was cultured in pleuropneumonia-like organism (PPLO) medium (BD Company, MD, USA) containing 10% horse serum (HyClone, UT, USA) at 37 °C. To determine the titre of *M. bovis* NX2, tenfold serial dilutions were spotted on PPLO agar plates, and the CFU/mL values were measured after 5 days of incubation at 37 °C And 5% CO_2_.

### Cell culture

BoMacs and MAC-T cells, generously provided by Prof. Aizhen Guo (Huazhong Agricultural University, China), were grown in RPMI 1640 (Gibco, USA) and DMEM/F-12 (Pricella, Wuhan, China) media, respectively, And incubated at 37 °C or 38.5 °C, respectively in 5% CO_2_. Both media were supplemented with 10% FBS (Pricella, Wuhan, China) And 1% penicillin/streptomycin before use.

### Cell viability assay

An MTT assay kit was used to investigate whether *M. bovis* NX2 influenced the viability of MAC-T cells. After 5 × 10^3^ MAC-T cells were seeded into each well of 96-well plates, they were incubated for 16 h. Then, cell infection was performed with *M. bovis* NX2 at different multiplicities of infection (5, 10, 20, 30, 50, And 100), after which cells were incubated for 24 h at 37 °C in 5% CO_2_. MTT solution (20 µL), prepared according to the instructions provided, was then added to each well, And after the plates were incubated for 4 h, the purple formazan in each well was dissolved in 100 μL of DMSO. Absorbance readings at 560 nm were recorded with a microplate reader (Bio-Rad Laboratories, USA).

### Extraction and quantification of EVs

After 1 × 10^7^ MAC-T cells were inoculated into 150-mm dishes, the cells were incubated for 16 h. PBS solution was then used to wash the cells three times before the addition of DMEM/F-12 (containing 10% exosome-depleted foetal bovine serum) with or without *M. bovis* NX2 (MOI: 10). After 24 h of culture, the cells were subjected to three rounds of centrifugation, first for 10 min at 300 × *g*, followed by 10 min at 2000 × *g* And finally 30 min at 10 000 × *g*. The resulting supernatant was then filtered through a 0.22/0.10-μm membrane, And after centrifugation at 120 000 × *g*, ultracentrifugation was performed for 70 min at 4 °C using a P70AT rotor (Hitachi, Tokyo, Japan). In addition, 10 μL of isolated EVs was incubated on PPLO plates to ensure the absence of interference from *M. bovis* NX2. After the resulting EVs were resuspended in 100 μL of PBS, the total protein content of each sample was determined with a BCA protein assay kit (Beyotime, Shanghai, China), and the remainder of the sample was kept for subsequent experiments.

### Nanoparticle tracking analysis (NTA)

EV samples (Ctrl-EVs and *M. bovis* NX2-EVs) were diluted with PBS at a ratio of 1:1000, and their size and distribution were subsequently determined by dynamic light scattering (DLS) using a nanoparticle size and zeta potential analyser (Anton Paar, Hohenbrugg, Austria).

### Transmission electron microscopy (TEM)

After placing 10 µL of Ctrl-EVs and *M. bovis* NX2-EVs on a grid, the samples were air-dried for 15 min at 37 °C to precipitate the EVs. This was followed by negative staining with 1% phosphotungstic acid for 30 s, followed by air-drying at room temperature. Then, the morphology of the EVs was observed under an HT7700 transmission electron microscope (Hitachi, Tokyo, Japan) operated at a voltage of 120 kV.

### EV fluorescent labelling and cellular uptake

BoMacs (3 × 10^5^ cells/mL) were seeded onto sterile coverslips or into 12-well plates, after which Ctrl-EVs and *M. bovis* NX2-EVs were labelled with the lipophilic fluorescent dye DID (1:200; Invitrogen, USA) for 30 min at 37 °C. DID-labelled EVs (50 μg/mL) were then incubated with BoMacs for 12 h before the cells were washed three times with PBS. The cells were then fixed for 10 min with 4% paraformaldehyde, and after the washing step was repeated, the nuclei were labelled with DAPI for visualization by confocal microscopy (Leica TCS SP8, Germany). In addition, BoMacs pretreated with or without endocytosis inhibitors for 6 h were incubated with DID-labelled EVs (50 μg/mL) for Another 12 h. The cells were then collected for fluorescence detection by flow cytometry (Sony MA900, Tokyo, Japan).

### Western blotting

BoMacs were incubated with RIPA buffer (Beyotime) for total protein extraction, and a BCA protein assay kit (Beyotime) was subsequently used to determine the amount of protein and EVs in the cellular extracts. After the extracted proteins (20 µg) were mixed with 6 × loading buffer (Takara Bio, Tokyo, Japan), SDS‒PAGE was performed on a 10% gel, after which the separated proteins were transferred to PVDF membranes (Millipore, Darmstadt, Germany). These membranes were then blocked for 1 h at 37 °C using 5% skim milk prior to overnight incubation at 4 °C with the following primary antibodies: anti-TSG101 (1:1000, ProteinTech, Wuhan, China); anti-calnexin (1:1000, ProteinTech), anti-TNF-α (1:1000, ProteinTech), anti-TNF-α (Mus, 1:20 000, ABclonal, China), anti-NOX [29], anti-GAPDH (1:1000, ProteinTech), anti-CD63 (1:1000, System Biosciences, Beijing, China), anti-CD81 (1:1000, System Biosciences), anti-ARCN1 (1:1000, ProteinTech), anti-MAPRE1 (1:1000, Affinity, Jiangsu, China), anti-APLP2 (1:2000, ProteinTech), anti-JAG1 (1:1000; ProteinTech), and purified polyclonal rabbit anti-JCHAIN antibody (1:1000, AtaGenix, Wuhan, China). This was followed by incubation for 1 h at room temperature with HRP-conjugated Affinipure goat anti-mouse and goat anti-rabbit IgG (H + L) (1:10 000, ProteinTech) secondary antibodies. The protein bands were subsequently developed with a chemiluminescence kit (Advansta) prior to visualization with An Amersham Imager 600 (Cytiva, USA). The intensity of each protein band was assessed against that of GAPDH and analysed with ImageJ (version 1.46).

### Reverse transcription‒quantitative PCR (RT‒qPCR)

TRIzol reagent (Invitrogen, CA, USA) was used to extract total RNA before reverse transcription into cDNA using the HiScript III RT SuperMix for qPCR (+ gDNA wiper) Kit (Vazyme, Nanjing, China). In this case, the reaction was performed for 15 min at 37 °C And for 5 s at 85 °C. This was followed by qRT–PCR, which was performed with the ChamQ Universal SYBR qPCR Master Mix Kit (Vazyme) under the following reaction conditions: initial denaturation for 30 s at 95 °C, followed by 40 cycles of 10 s of denaturation at 95 °C And 30 s of extension at 60 °C. The results were normalized to those of GAPDH, And the 2^−∆∆Cq^ method was subsequently used to determine the level of relative expression. The primers selected for this experiment are listed in Table 1 and were synthesized by Sango Biotechnology (Shanghai).

**Table 1:** **Sequences of primers used for qRT‒PCR**

Gene	Primer sequence (5′-3′)
IL-1β F (Bovine)	GTCATCTTCGAAACGTCCTCC
IL-1β R (Bovine)	TCCTCTCCTTGCACAAAGCTC
IL-6 F (Bovine)	ACCCCAGGCAGACTACTTCT
IL-6 R (Bovine)	CCCAGATTGGAAGCATCCGT
IL-8 F (Bovine)	GAAGAGAGCTGAGAAGCAAGATCC
IL-8 R (Bovine)	ACCCACACAGAACATGAGGC
TNF-α F (Bovine)	CTCCATCAACAGCCCTCTGG
TNF-α R (Bovine)	GAGGGCATTGGCATACGAGT
GAPDH F (Bovine)	TGGTGAAGGTCGGAGTGAAC
GAPDH R (Bovine)	ATGGCGACGATGTCCACTTT
IL-1β F(Mus)	TCGCTCAGGGTCACAAGAAA
IL-1β R (Mus)	CATCAGAGGCAAGGAGGAAAAC
IL-6 F (Mus)	ACAAGTCGGAGGCTTAATTACACAT
IL-6 R (Mus)	TTGCCATTGCACAACTCTTTTC
TNF-α F (Mus)	AGGCTGCCCCGACTACGT
TNF-α R (Mus)	GACTTTCTCCTGGTATGAGATAGCAAA
GAPDH F (Mus)	TCCCACTCTTCCACCTTCGA
GAPDH R (Mus)	AGTTGGGATAGGGCCTCTCTT

### Immunofluorescence staining

BoMacs (2 × 10^5^ cells/mL) were seeded onto sterile coverslips prior to incubation for 24 h with Ctrl-EVs and *M. bovis* NX2-EVs. PBS was then used to wash the slides three times (1 min each), And after 15 min of cell fixation at room temperature using 4% paraformaldehyde, 0.1% Triton X-100 was added for 15 min at 37 °C for cell permeabilization. Blocking was then performed for 1 h using 10% BSA prior to overnight incubation at 4 °C with the following primary antibodies: anti-TNF-α (1:200, Proteintech), anti-IL-1β (1:200, Proteintech) and anti-IL-6 (1:200, Affinity). After the cells were washed with PBS five times, they were incubated for 30 min at 37 °C with Alexa Fluor 594-conjugated goat anti-rabbit IgG (H + L) (1:1000, Thermo Fisher) And Alexa Fluor 488-conjugated goat anti-mouse IgG (H + L) (1:2000, Thermo Fisher). This was followed by nuclear counterstaining with DAPI (Thermo Fisher) or Hoechst (Beyotime) before visualization with a confocal laser scanning microscope (Leica TCS SP8).

### Preparation of BMDMs

BMDMs were isolated And harvested from the tibiae of 6–8-week-old wild-type C57BL/6J mice. Cells were washed once with PBS and then cultured in low-glucose DMEM (Gibco, USA) supplemented with 10% FBS (Pricella, Wuhan, China), 1% penicillin/streptomycin (Invitrogen, USA), And 20 ng/mL mouse macrophage colony-stimulating factor (MCE, USA). The cells were subsequently incubated at 37 °C in 5% CO_2_ for 7 days. Then, the adherent cells were washed and harvested with trypsin (Invitrogen, USA).

### Isolation of bovine peripheral blood mononuclear cells (PBMCs) and differentiation into macrophages

Blood samples (50 mL from each animal) were obtained from 3 clinically healthy animals with no history of *M. bovis* infection. A bovine peripheral blood monocyte isolation kit (Solarbio, China) was used to isolate PBMCs. Red blood cell lysis buffer (Beyotime) was used to lyse erythrocytes. Cells were washed twice with PBS containing 1 mM EDTA (Beyotime) and then cultured in RPMI-1640 (Gibco, USA) supplemented with 10% FBS (Pricella), 1% penicillin/streptomycin (Invitrogen, USA), 10 μg/L insulin (MCE, USA), growth-promoting compounds (1 mg/L progesterone, 0.05% lactalbumin And 0.05% a-lactose) And 20 ng/mL bovine granulocyte macrophage-colony stimulating factor (MCE, USA) [30]. The cells were subsequently incubated at 38.5 °C in 5% CO_2_ for 7 days [31]. Then, the adherent cells were washed and harvested with trypsin (Invitrogen, USA).

### Flow cytometry

For all experiments, cell surface staining was performed in the dark in PBS at 4 °C for 1 h. The cells were then washed twice with PBS and incubated with antibodies against surface markers, including FITC-conjugated anti-CD11b (0.2 mg/mL, Proteintech) and FITC-conjugated anti-CD14 antibody (1:10, Bio-Rad, CA, USA). Cells were collected for flow cytometry and data analysis using a flow cytometer (Sony MA900, Tokyo, Japan) and FlowJo-V10 software.

### RNA-seq and genome-wide transcriptome analysis

RNA-seq was performed by Novogene (Beijing, China). Ctrl-EVs and *M. bovis* NX2-EVs were incubated with BoMacs (1 × 10^6^ per well) for 12 And 24 h before TRIzol reagent was used to extract total RNA for RNA sequence analysis (three biological replicates per set). Oligo dT magnetic beads were then used for RNA purification before generating libraries with the NEBNext Ultra RNA Library Prep Kit for Illumina (NEB, USA). In this case, indexing codes were also added to the sequences for each sample. For each Library, quantification was performed using a Qubit 2.0 fluorometer, And after being diluted to 1 ng/μL, its quality was assessed with An Agilent 2100 bioanalyzer. For data analysis, the raw sequences were first subjected to quality control, which involved filtering as well as checking for sequencing errors and the distribution of the GC content to yield clean reads. The DESeq2 package was subsequently used to analyse differential expression, with significance considered at *p* values adjusted using the Benjamini and Hochberg method. In addition, the clusterProfiler R package was used for KEGG pathway enrichment analyses.

### LC‒MS/MS and proteomic analysis

Technical support for the proteomic analysis was provided by Applied Protein Technology (Shanghai, China). Ctrl-EVs and *M. bovis* NX2-EVs (three samples per group) were separately analysed by LC‒MS/MS (DIA mode, Astral Mass Spectrometer) prior to protein identification by line DIA. Significantly differentially expressed proteins were then screened using a* p* value of < 0.05 (*t* test) and an expression fold change (FC) of > 1.5-fold as thresholds, after which the subcellular localization of these differentially expressed proteins was analysed using CELLO, a software for predicting subcellular structures. This was followed by GO functional annotation and KEGG pathway analysis of all significantly differentially expressed proteins using Blast2Go and KOBAS (version 3.0) software, respectively.

### Immunocolloid electron microscopy

A suspension of EVs was purified as described above, And after being mixed with 4% paraformaldehyde (1:1), the resulting mixture was dropped onto a nickel mesh with a carbon film And allowed to stand for 1 h. PBS was then used to wash the EVs three times before they were blocked for 30 min using 1% BSA. The grids were subsequently incubated for 2 h at 4 °C with appropriate dilutions of the primary antibodies against ARCN1 (1:1000, ProteinTech), MAPRE1 (1:1000, Affinity), JAG1 (1:1000, ProteinTech), TSG101 (1:1000, ProteinTech) and JCHAIN (1:1000, AtaGenix), and after repeating the washing step (3 min each time), the grid was floated for 2 h at room temperature on Anti-rabbit IgG, Anti-mouse IgG, and secondary antibody droplets containing 10-nm gold particles (AURION, Hatfield, PA). The washing step was repeated again with PBS (3 min each time), And after the samples were fixed for 5 min with 2% glutaraldehyde, a final wash was performed. The grids were then air-dried and stained with uranyl acetate prior to visualization using a Tecnai Bio Twin transmission electron microscope (FEI, Hillsboro, Oregon), with the images captured using an AMT CCD camera (Advanced Microscopy Techniques, Danvers, Massachusetts).

### Sequencing of EV-derived miRNAs

EV-derived miRNA sequencing was performed by Applied Protein Technology (Shanghai, China). Total RNA was extracted from Ctrl-EVs and *M. bovis* NX2-EVs (three samples per group) with TRIzol reagent (Invitrogen) before An Agilent 2100 Bioanalyzer (Agilent, USA) was used to assess the concentration and purity of the RNA. Small RNA libraries were subsequently constructed using an Illumina small RNA sample preparation kit, and after their quality and quantity were checked with an Agilent High Sensitivity DNA Kit and a Quant-iT PicoGreen dsDNA Assay Kit, respectively, sequencing was performed. Differentially expressed miRNAs were eventually identified with the edgeR algorithm using a *p* value of < 0.05 and a log_2_ (FC) of > 1 as thresholds after the data were normalized with Agilent Gene Spring software.

### EV-derived miRNA RT‒qPCR

The miRNeasy Mini Kit (QIAGEN, USA) was used to isolate miRNA from Ctrl-EVs and *M. bovis* NX2-EVs. This was followed by reverse transcription and qRT‒PCR, which were performed according to the instructions provided for the All-in-One miRNA qRT‒PCR Detection Kit 2.0 (GeneCopoeia, USA). The 2^−∆∆Cq^ method was then used to determine the relative expression levels, and the results were normalized to those of U6. The primers selected for the amplification process are listed in Table 2 and were synthesized by Sango Biotechnology (Shanghai).

**Table 2:** **Primer sequences for miRNA qRT‒PCR**

Gene	Primer sequence (5′-3′)
Universal Primer R	CGCTGTCAACGATACGCTACGTAAC
bta-miR-149-5p F	ATATCTGGCTCCGTGTCTTCACTCC
bta-miR-1307 F	TTATAATTATACTCGGCGTGGCGTCG
bta-miR-12043 F	ATTACCTCCAGGGCTAGGAGGTG
bta-miR-11987 F	CGAGGAATCTCTGGTGGAGGT
U6 F	CGAACGCTTCACGAATTTGCGT
U6 R	CTCGCTTCGGCAGCACA

### Statistical analyses

For each measurement, the results were obtained from at least three independent experiments, are presented as the mean ± standard error of the mean (SEM), And were Analysed in GraphPad Prism version 6.0 (GraphPad Software, CA, USA). For parametric data, multiple comparisons of populations of equal variance were performed using one-way ANOVA and post hoc tests (Tukey’s test), whereas pairwise comparisons were performed using two-tailed Student’s *t* tests. In this case, statistical significance was indicated by *p* < 0.05, and *p* values < 0.05, 0.01, And 0.001 are marked as *, **, and *** in the figures, respectively.

## Results

### Characterization of EVs secreted by MAC-T cells and induced by *M. bovis* NX2 infection

**Figure 1:**
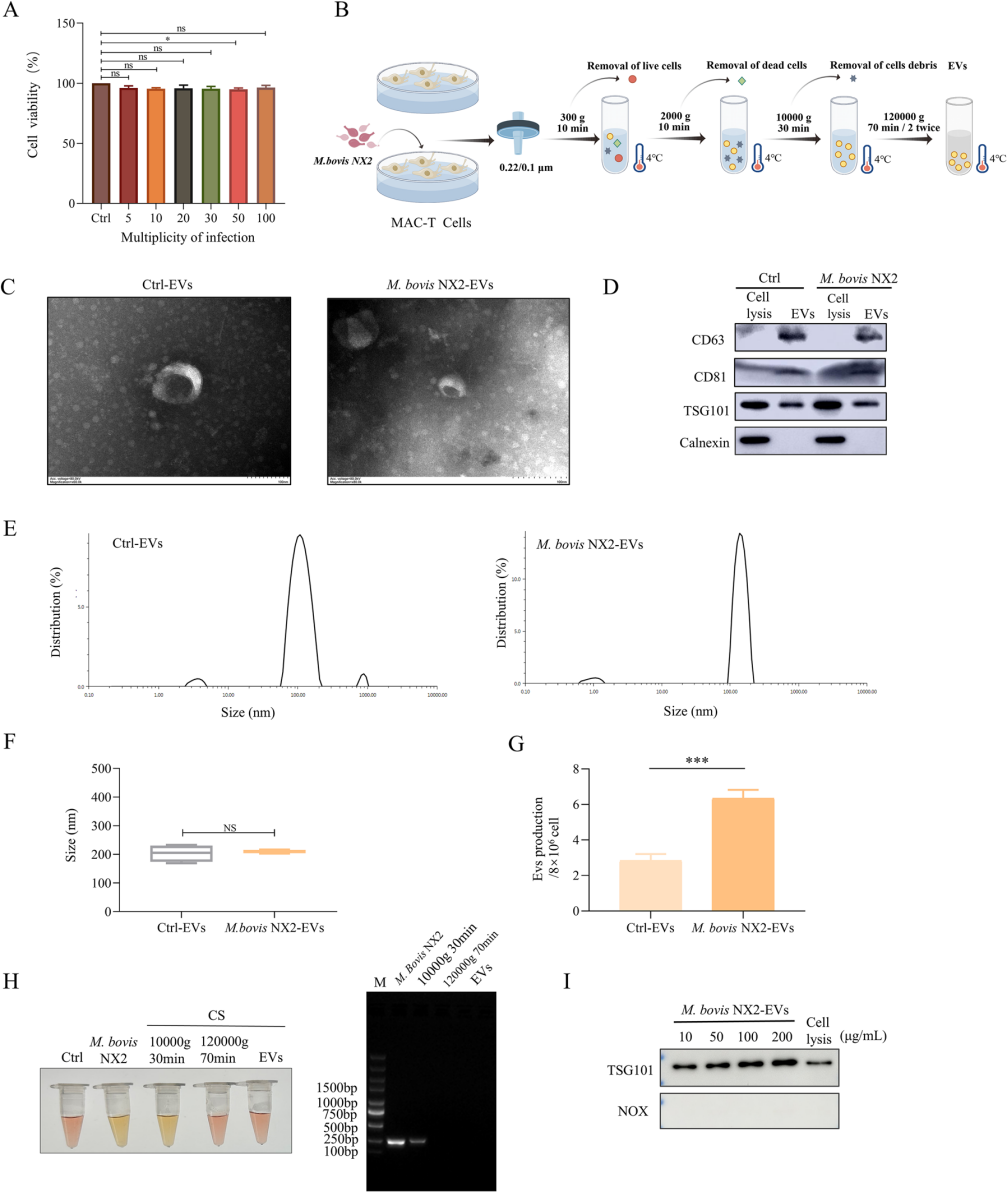
**Characterization of Ctrl-EVs and** ***M. bovis*** **NX2-EVs**. **A** MTT assay to assess the viability of MAC-T cells infected with *M. bovis* NX2 for 24 h at different MOIs (5, 10, 20, 30, 50, And 100). **B** Schematic diagram of the Ctrl-EV and *M. bovis* NX2-EV separation process. **C** Representative TEM images of Ctrl-EVs and *M. bovis* NX2-EVs. **D** Semiquantitative analysis of CD63, CD81 and TSG101 along with the negative control calnexin by western blotting. **E**, **F** Size distribution of Ctrl-EVs and *M. bovis* NX2-EVs analysed by a nanoparticle tracking analyser. **G** Concentrations of Ctrl-EVs and *M. bovis* NX2-EVs as determined by the BCA assay. Scale bar: 100 nm. **H** Ctrl, *M. bovis* NX2 culture media. *M. bovis* NX2 was cultured as a positive control. CS (cell supernatants; 10 000 × *g* for 30 min). The supernatants of *M. bovis* NX2-infected MAC-T cells were centrifuged at 10 000 × *g* for 30 min to isolate the EVs. After centrifugation at 120 000 × *g* for 70 min, the supernatants of *M. bovis* NX2-infected MAC-T cells were centrifuged at 120 000 × *g* for 70 min to isolate the EVs. EVs, EVs from *M. bovis* NX2-infected MAC-T cells. These components were added to PPLO And cultured for 7–10 days. Detection of *M. bovis* NX2 in the process of EV isolation by PCR. **I** The abundance of the TSG101 and NOX proteins was determined by western blotting. The results are presented as the mean ± SEM from three independent experiments, and two-tailed Student’s *t* tests were used to analyse the data shown in (**A**), (**F**) and (**G**); ***p < 0.001 indicates a statistically significant difference; ns indicates no difference.

EVs derived from infected cells were extracted without cell debris to determine their role in regulating the host inflammatory response. For this purpose, MAC-T cells were first infected with *M. bovis* for 24 h at different MOIs (5, 10, 20, 30, 50, And 100), And the MTT assay revealed that the viability of infected And uninfected cells did not significantly differ. Hence, an MOI of 10 was selected for subsequent experiments based on published literature (Figure 1A). EVs derived from *M. bovis*-infected and uninfected MAC-T cells and designated *M. bovis* NX2-EVs and Ctrl-EVs, respectively (Figure 1B), were characterized using TEM, DLS, western blotting and BCA analysis. Both types of EVs exhibited a cup-shaped morphology with a bilayered-membrane structure (Figure 1C) and expressed CD63, CD9 and TSG101 but not the cytoplasmic protein calnexin (Figure 1D). DLS analysis further revealed that the average diameters of the Ctrl-EVs and *M. bovis* NX2-EVs were 169.17 nm And 188.80 nm, respectively (Figures 1E and F). Therefore, the findings indicated that the two types of EVs were not significantly different in terms of size distribution or morphological characteristics. Interestingly, compared with that in the uninfected group, the concentration of EVs was greater in the group of MAC-T cells infected with *M. bovis* (Figure 1G). Furthermore, the absence of any potential interference from *M. bovis* NX2 in the EVs was verified through mycoplasma culture and PCR, and the results confirmed the absence of the organism from the extracted EVs (Figure 1H) as well as successful EV isolation for subsequent experiments. In addition, we detected the presence of NOX (a marker of *M. bovis* EVs) in *M. bovis* NX2-EVs (10, 50, 100 And 200 μg/mL) by western blotting. The *M. bovis* adhesion-associated protein NOX was not detected in *M. bovis* NX2-EVs (Figure 1I). Finally, we tested whether the survival of *M. bovis* NX2 would be affected in DMEM/F-12 supplemented with 10% FBS. The CFU results indicated that the MAC-T-cell culture media maintained live *M. bovis* NX2 for up to 12 h of incubation, but when the incubation time was extended to 24 h, the viability of *M. bovis* NX2 decreased slightly (Additional file 1).

### Uptake of EVs through endocytosis and megalocytosis in BoMacs

**Figure 2:**
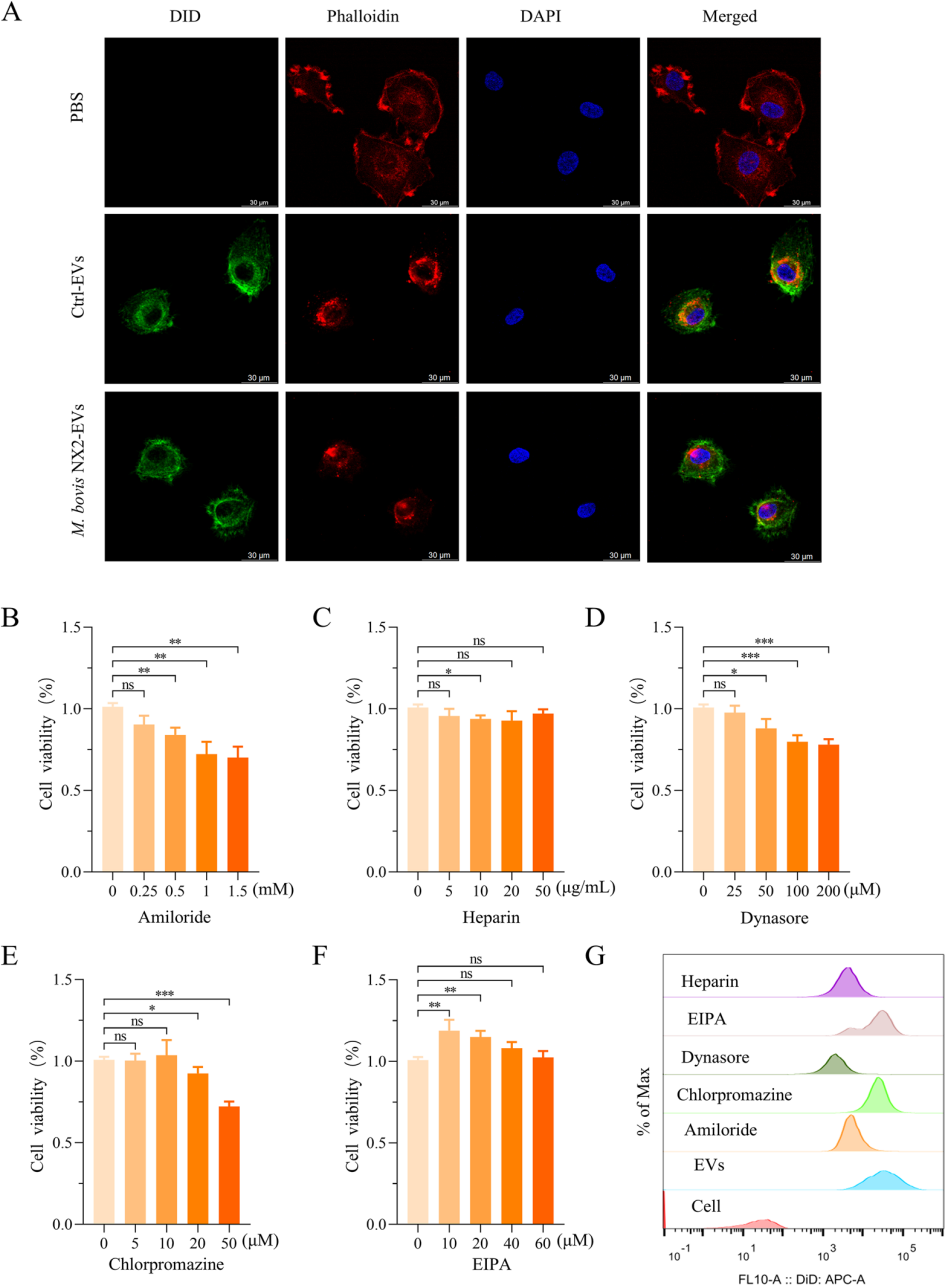
**Reduced uptake of EVs by BoMacs using endocytosis and megalocytosis inhibitors**. **A** Representative confocal images of Ctrl-EVs and *M. bovis* NX2*-*EVs after incubation with BoMacs for 24 h. DAPI-labelled nuclei (blue), phalloidin-labelled cytoskeleton (red), and DID-labelled Ctrl-EVs and *M. bovis* NX2*-*EVs (green). Scale bar: 30 μm. MTT assays were performed to assess the viability of BoMacs pretreated with different concentrations of amiloride (**B**), heparin (**C**), Dynasore (**D**), chlorpromazine (**E**) and EIPA (**F**) for 12 h. (**G**) Average intensity of DID fluorescence in BoMacs, as determined by flow cytometry, after 6 h of pretreatment with heparin (20 μg/mL), Dynasore (25 μM), amiloride (0.25 mM), chlorpromazine (5 μM) and EIPA (40 μM) and subsequent incubation with DID-labelled *M. bovis* NX2-EVs for 12 h. The results are presented as the mean ± SEM from three independent experiments, and two-tailed Student’s *t* tests were used to analyse the data shown in (**B**–**F**); *p > 0.05, **p < 0.01, and ***p < 0.001 indicate a significant difference; ns indicates no difference.

EVs can be taken up by receptor cells through different mechanisms that are dependent on the type of recipient cell and the source of the EV. To confirm the uptake of MAC-T-derived EVs by BoMacs, Ctrl-EVs and *M. bovis* NX2-EVs were labelled with fluorescent DID prior to 24 h of incubation with BoMacs. Immunofluorescence results subsequently revealed the presence of the fluorescent dye in the cytoplasm, thus confirming that all the EVs were internalized by the BoMacs (Figure 2A). The mechanisms involved in the uptake of the EVs were then investigated by first treating the cells for 12 h with different concentrations of the endocytosis inhibitors heparin (0, 5, 10, 20, And 50 μg/mL) and Dynasore (0, 25, 50, 100, And 200 μM) as well as the megalocytosis inhibitors amiloride (0, 0.25, 0.5, 1, And 1.5 mM), chlorpromazine (0, 5, 10, 20, And 50 μM) and EIPA (0, 10, 20, 40, And 60 μM) to determine their cytotoxicity at different concentrations. Overall, heparin and EIPA had less effects on cell viability, whereas in the case of cytochalasin D, amiloride and chlorpromazine, cell viability was dependent on the concentration used (Figures 2B–F). Based on the results, BoMacs were pretreated with 20 μg/mL heparin, 25 μM Dynasore, 0.25 mM amiloride, 5 μM chlorpromazine And 40 μM EIPA for 6 h, after which they were incubated with DID-labelled *M. bovis NX2*-EVs for 12 h before their EV uptake was assessed by flow cytometry. These inhibitors significantly reduced the uptake of EVs by BoMacs, with the endocytosis inhibitors heparin And Dynasore accounting for 80% of the inhibition (Figure 2G). Hence, while two pathways are involved in the internalization of EVs by BoMacs, endocytosis appears to be the main pathway involved in *M. bovis* NX2-EV-mediated communication between MAC-T cells and BoMacs.

### *Mycoplasma bovis* NX2-EVs induce an inflammatory response in BoMacs

**Figure 3:**
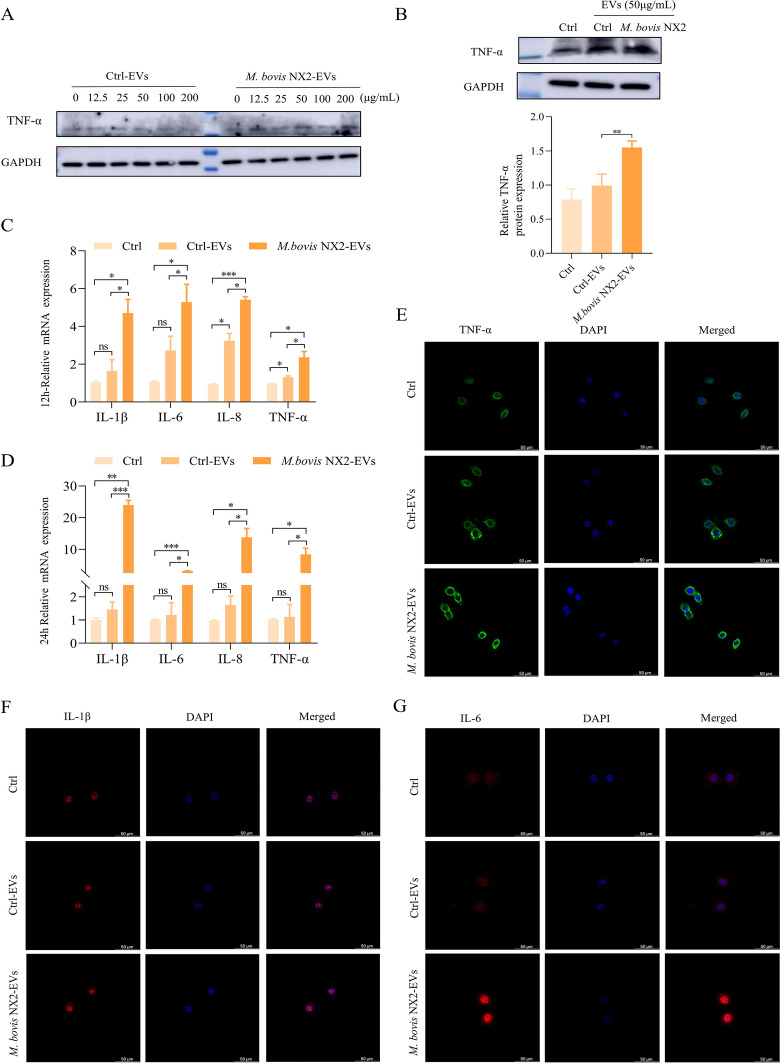
**Effects of Ctrl-EVs and** ***M. bovis*** **NX2-EVs on the inflammatory response of BoMacs**. **A**, **B** western blotting was used to measure TNF-α levels after BoMacs were incubated with 0, 12.5, 25, 50, 100 And 200 μg/mL Ctrl-EVs and *M. bovis* NX2-EVs for 24 h. **C**, **D** Expression levels of TNF-α, IL-8, IL-6 and IL-1β, as determined by qRT‒PCR, after BoMacs were incubated for 12 or 24 h with 50 μg/mL Ctrl-EVs and *M. bovis* NX2-EVs. Representative immunofluorescence images of BoMacs pretreated with anti-TNF-α antibody (green) (**E**), anti-IL-1β antibody (red) (**F**), and anti-IL-6 antibody (red) (**G**) as well as DAPI to label nuclei (blue) before 24 h of incubation with 50 μg/mL Ctrl-EVs and *M. bovis* NX2-EVs. Scale bar: 50 μm. The results are presented as the mean ± SEM of three independent experiments; one-way ANOVA and Tukey’s test were used to analyse the data shown in (**C**) and (**D**), while a two-tailed Student’s *t* test was used to analyse the data shown in (**B**); *p > 0.05, **p < 0.01, and ***p < 0.001 indicate a significant difference; ns indicates no difference.

BoMacs were incubated for 24 h with 0, 12.5, 25, 50, 100 And 200 μg/mL EVs to determine whether *M. bovis* NX2-EVs could induce An inflammatory response in the cells. Western blotting subsequently revealed that 50 μg/mL *M. bovis* NX2-EVs could significantly increase the expression of the proinflammatory cytokine TNF-α; hence, this concentration was selected for subsequent experiments (Figures 3A and B). Since the body temperature of cattle is usually approximately 38.5 °C and has been verified to impact the bovine immune response to *M. bovis* [31], we next investigated whether body temperature could influence the expression of TNF-α in BoMacs treated with *M. bovis* NX2-EVs. The results indicated that *M. bovis* NX2-EVs increased the expression of TNF-α in BoMacs at 38.5 °C (Additional file 2). BoMacs were then incubated with 50 μg/mL Ctrl-EVs and *M. bovis* NX2-EVs for 12 And 24 h before the expression of proinflammatory cytokines was measured by qRT‒PCR. Compared with the Ctrl and Ctrl-EVs, *M. bovis* NX2-EVs elicited significantly higher levels of TNF-α, IL-8, IL-6 and IL-1β (Figures 3C and D), and these results were further confirmed by immunofluorescence, which revealed the significantly increased fluorescence intensity for IL-6, IL-1β and TNF-α in *M. bovis* NX2-EVs (Figures 3E–G). Taken together, these results suggest that *M. bovis* NX2 infection of MAC-T cells may induce an inflammatory response in BoMacs through the release of EVs.

### *Mycoplasma bovis* NX2-EVs induce an inflammatory response in BMDMs and bovine monocyte-derived macrophages

**Figure 4:**
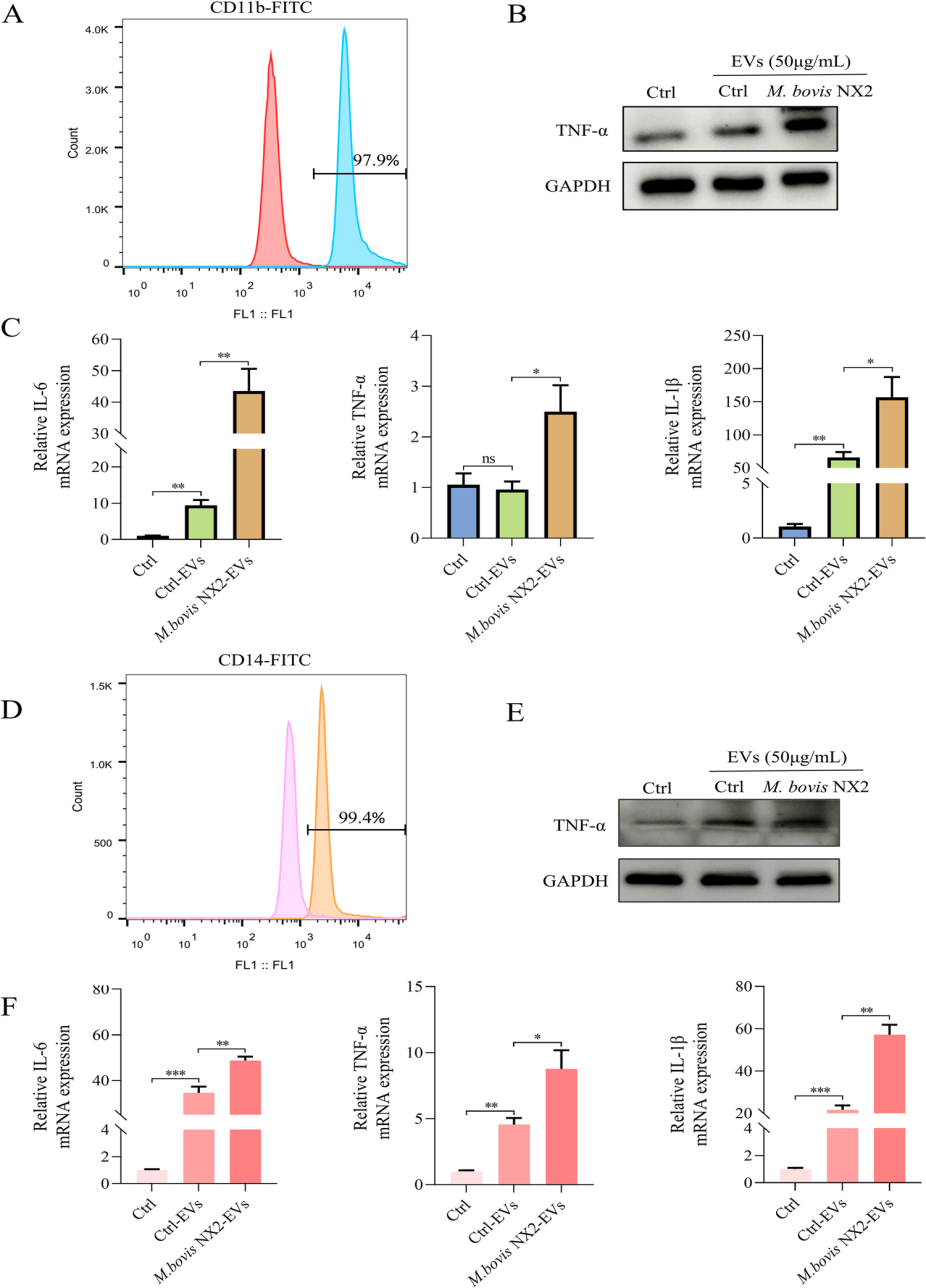
***Mycoplasma bovis*** **NX2-EVs induce an inflammatory response in monocyte-derived macrophages**. **A** Verification of mouse bone marrow-derived macrophages (BMDMs) by flow cytometry. **B** Western blotting was used to measure TNF-α levels in BMDMs after they were incubated with 50 μg/mL Ctrl-EVs or *M. bovis* NX2-EVs for 24 h. **C** Expression levels of TNF-α, IL-6 and IL-1β, as determined by qRT‒PCR, after BMDMs were incubated for 24 h with 50 μg/mL Ctrl-EVs and *M. bovis* NX2-EVs. **D** Verification of bovine monocyte-derived macrophages by flow cytometry. **E** western blotting was used to measure TNF-α levels in bovine monocyte-derived macrophages after they were incubated with 50 μg/mL Ctrl-EVs or *M. bovis* NX2-EVs for 24 h. **F** Expression levels of TNF-α, IL-6 and IL-1β, as determined by qRT‒PCR, after bovine monocyte-derived macrophages were incubated with 50 μg/mL Ctrl-EVs or *M. bovis* NX2-EVs for 24 h. The results are presented as the mean ± SEM of three independent experiments; Student’s *t* test was used to analyse the data; *p > 0.05, **p < 0.01, and *** *p* < 0.001 indicate a significant difference; ns indicates no difference.

Although BoMacs a widely used bovine macrophage cell line, primary cells better mimic in vivo conditions than cell lines do. Therefore, we isolated bovine monocyte-derived macrophages and BMDMs for further study. To determine the purity of the BMDMs, the cells were stained with an anti-CD11b antibody. Flow cytometry analysis revealed that the harvested adherent cells contained > 95% CD11b-labelled macrophages (Figure 4A). BMDMs were then incubated with 50 μg/mL Ctrl-EVs and *M. bovis* NX2-EVs for 24 h before the expression of proinflammatory cytokines was detected by western blotting and qRT‒PCR. As expected, compared with Ctrl-EVs, *M. bovis NX2*-EVs significantly promoted the expression of IL-6, IL-1β and TNF-α (Figures 4B and C). To identify bovine monocyte-derived macrophages after isolation and differentiation, the cells were labelled with an anti-CD14 antibody. Flow cytometry analysis revealed that the harvested adherent cells contained > 95% CD14-labelled macrophages (Figure 4D). Subsequently, bovine monocyte-derived macrophages were treated with 50 μg/mL Ctrl-EVs and *M. bovis* NX2-EVs for 24 h. Similarly, *M. bovis* NX2-EVs increased the levels of proinflammatory cytokines (IL-1β, IL-6 and TNF-α) in bovine monocyte-derived macrophages (Figures 4E and F). Taken together, these results strongly suggest that EVs derived from *M. bovis* NX2-infected MAC-T cells can induce inflammatory responses in macrophages.

### Differential gene enrichment and analysis of the *M. bovis* NX2-EV-induced inflammatory response in BoMACs

**Figure 5:**
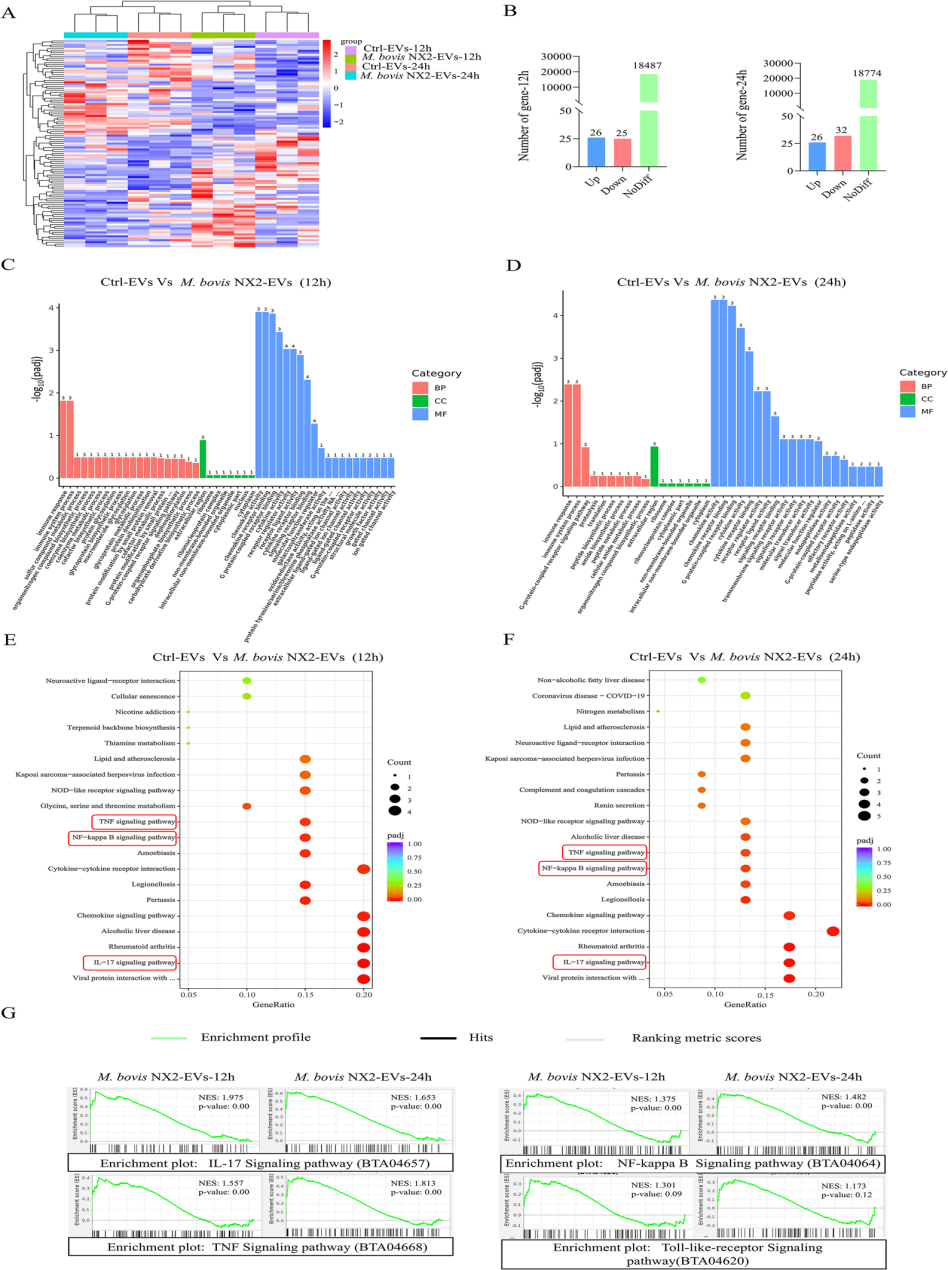
**Effects of Ctrl-EVs and** ***M. bovis*** **NX2-EVs on the inflammatory response of BoMacs**. **A** Heatmap showing differentially expressed genes (DEGs) in response to different treatments. **B** Bar graph showing the number of DEGs in different treatment groups. **C**, **D** GO enrichment analysis of all significant DEGs, with BP (biological process), CC (cellular component) and MF (molecular function) indicated by red, green and blue, respectively. **E**, **F** KEGG pathway enrichment analysis of DEGs. **G** Gene set enrichment analysis of the Toll-like receptor, NF-kappa B, TNF and IL-17 signalling pathways.

To further explore the differential gene expression induced by Ctrl-EVs and *M. bovis* NX2-EVs in BoMacs during the inflammatory response, transcriptome sequencing was performed after 12 And 24 h of treatment. After 12 h, 12 714 genes were common, whereas 233 And 194 genes were unique to BoMacs that had been treated with Ctrl-EVs and *M. bovis* NX2-EVs, respectively. Similarly, BoMacs showed 12,853 shared genes but 222 And 180 unique genes following 24 h of treatment with Ctrl-EVs and *M. bovis* NX2-EVs. A hierarchical cluster plot was then constructed to display the significantly differentially expressed genes (DEGs, adjusted *p* < 0.05, fold change > 1.5) in the different treatment groups (Figure 5A). Overall, it was found that the 12-h treatment with *M. bovis* NX2-EVs significantly upregulated 26 genes And downregulated 25 genes in BoMacs as opposed to treatment with Ctrl-EVs. Additionally, incubating BoMacs with *M. bovis* NX2-EVs for 24 h led to the significant upregulation of 26 genes, while 32 genes were downregulated (Figure 5B). GO enrichment Analysis subsequently revealed that after both 12 And 24 h of treatment, the DEGs were enriched in chemokine receptor binding, chemokine activity, immune system processes, immune response, signalling receptor binding, cytokine receptor binding, receptor regulator activity, cytokine activity and G protein-coupled receptor binding (Figures 5C and D). Similarly, KEGG enrichment analysis revealed that the main pathways in which the DEGs were involved included the rheumatoid arthritis, NF-kappa B, TNF, IL-17 and chemokine signalling pathways, as well as many other biological processes associated with cellular inflammatory responses (Figures 5E and F). Subsequent GSEA analysis showed that Toll-like-receptors, NF-kappa B, TNF and IL-17, which are associated with higher ENS scores and inflammatory pathways, were all enriched in *M. bovis* NX2-EV-treated cells (Figure 5G). The NF-kappa B pathway and Toll-like receptor signalling are crucial for inflammation and innate immunity because of their involvement in the release of inflammatory cytokines and in modulating responses. These findings suggest that *M. bovis* NX2-EVs induce inflammatory responses in BoMacs, potentially through the NF-kappa B pathway and Toll-like receptor signalling.

### Screening and validation of differentially expressed proteins in Ctrl-EVs and *M. bovis* NX2-EVs

**Figure 6:**
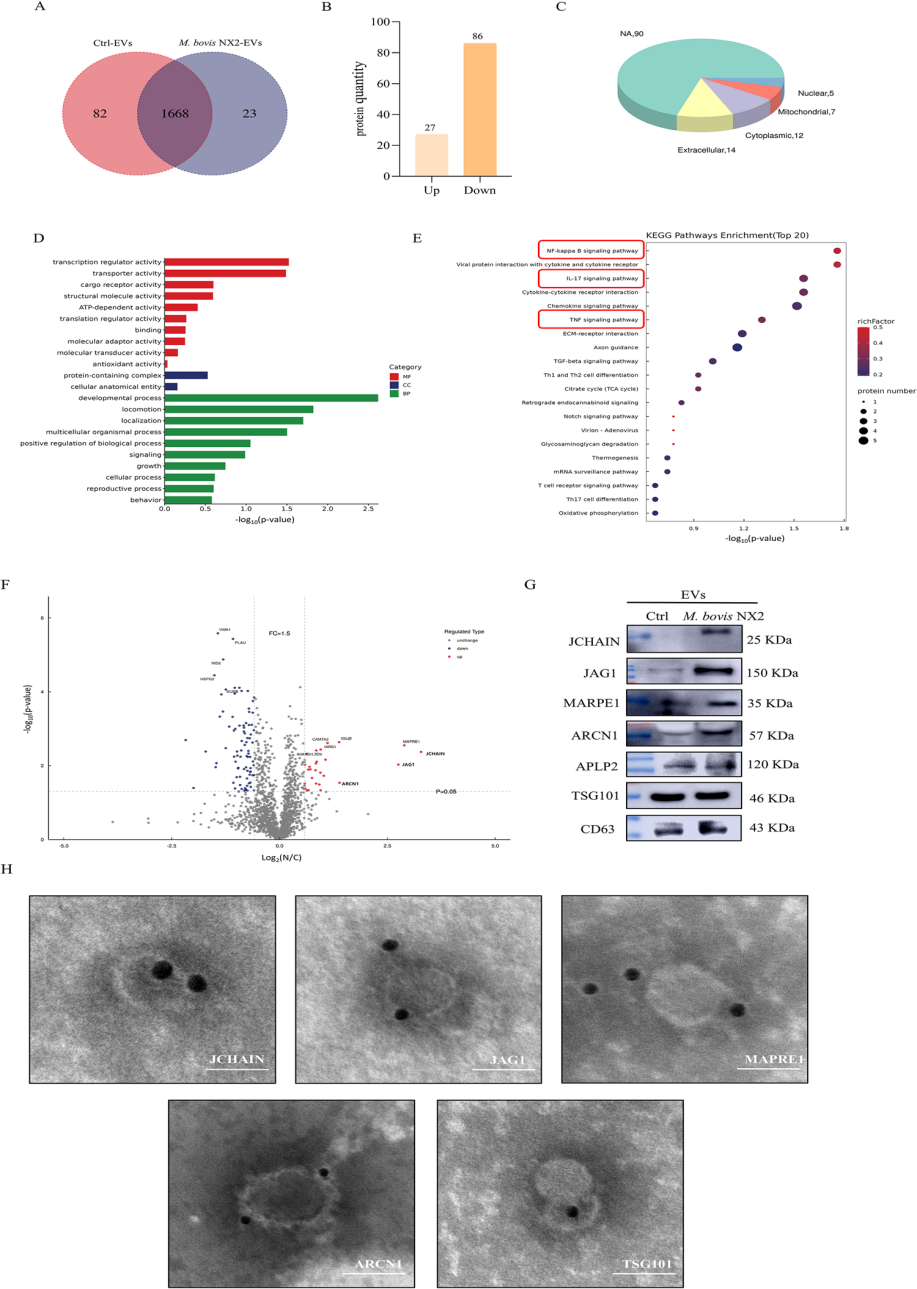
**Quantitative proteomic analysis of differentially expressed proteins in Ctrl-EVs and** ***M. bovis*** *NX2-EVs*. **A** Venn diagram showing common and unique proteins in Ctrl-EVs and *M. bovis* NX2-EVs. **B** Statistics on the identification and quantification of proteins in Ctrl-EVs and *M. bovis* NX2-EVs. **C** Histogram of the subcellular localization of differentially expressed proteins between *M. bovis* NX2-EVs and Ctrl-EVs. **D** GO enrichment analysis of all significantly differentially expressed proteins; BP (biological process), CC (cellular component) and MF (molecular function) are indicated by green, blue and red, respectively. **E** KEGG enrichment analysis of all significantly differentially expressed proteins. **F** Volcano diagram of differentially expressed proteins between *M. bovis* NX2-EVs and Ctrl-EVs; red and blue dots indicate significantly upregulated and downregulated proteins, respectively, whereas grey dots represent proteins whose expression was not significantly different. **G** The abundance of the JCHAIN, JAG1, MAPRE1, ARCN1, APLP2, TSG101 and CD63 proteins was determined by western blotting. **H** Representative TEM of EVs labelled with immunogold using anti-JCHAIN, anti-JAG1, anti-MAPRE1, anti-ARCN1 and anti-TSG101 Antibodies. Scale bar: 100 nm.

The above results indicated that *M. bovis* NX2-EVs induced An inflammatory response in BoMacs, And it was hypothesized that different components of EVs could be involved in the induction of that response. In this case, proteomic analyses revealed a total of 1773 proteins, of which 82 And 23 were unique to Ctrl-EVs and *M. bovis* NX2-EVs, respectively (Figure 6A). Interestingly, no proteins attributed to *M. bovis* were identified. Subsequent differential analyses revealed that compared with the Ctrl-EVs, the *M. bovis* NX2-EVs resulted in significant changes in the expression of 113 proteins (86 upregulated And 27 downregulated) (Figure 6B), with organelle localization analysis further indicating that these differential proteins were mostly localized in the cytoplasmic, nuclear, mitochondrial and extracellular spaces (Figure 6C). To better understand the functions and localization of the differential proteins as well as the biological pathways in which they were involved, GO functional annotation was performed. With respect to biological processes (BP), the proteins were enriched mainly in cell developmental processes, whereas in terms of cellular components (CC), protein-containing complexes were enriched the most. Finally, regarding molecular function (MF), the proteins were enriched mainly in transcriptional regulators (Figure 6D). This was followed by KEGG pathway enrichment analysis of the differentially expressed proteins, with the results highlighting significant enrichment of cytokine‒cytokine receptor interactions, chemokine and TNF signalling, IL-17, viral protein interactions with cytokines and cytokine receptors and NF-kappa B pathways (Figure 6E). Taken together, these findings suggest that the differentially expressed proteins in EVs may also be crucial for regulating inflammatory responses. In addition, the significant upregulation of the JCHAIN, JAG1, MAPRE1 and ARCN1 proteins was confirmed by western blotting, with APLP2, a nonsignificantly different protein, selected as the negative control. In this case, compared with the Ctrl-EVs, the *M. bovis* NX2-EVs exhibited significantly higher expression of the JCHAIN, JAG1, MAPRE1 and ARCN1 proteins, as shown in the results of the quantitative proteomic volcano plots (Figures 6F and G). However, CD63 and TSG101 expression was detected in both types of EVs. The EVs derived from *M. bovis* NX2-infected MAC-T cells were further characterized by electron microscopy with immunogold labelling to determine the localization of their differentially expressed proteins. The results revealed that JCHAIN, JAG1, MAPRE1 and ARCN1 were localized on the membrane of EVs, whereas the marker protein TSG101 was present in the cytoplasm (Figure 6H). These findings suggest that EVs released from *M. bovis* NX2-infected MAC-T cells are heterogeneous, and the involvement of their proteins in chemokine activity, cytokine receptor binding and the regulation of factors involved in transcriptional control further highlights their significance in inducing inflammatory responses in BoMacs.

### Differential miRNA expression profiles between Ctrl-EVs and *M. bovis* NX2-EVs

**Figure 7:**
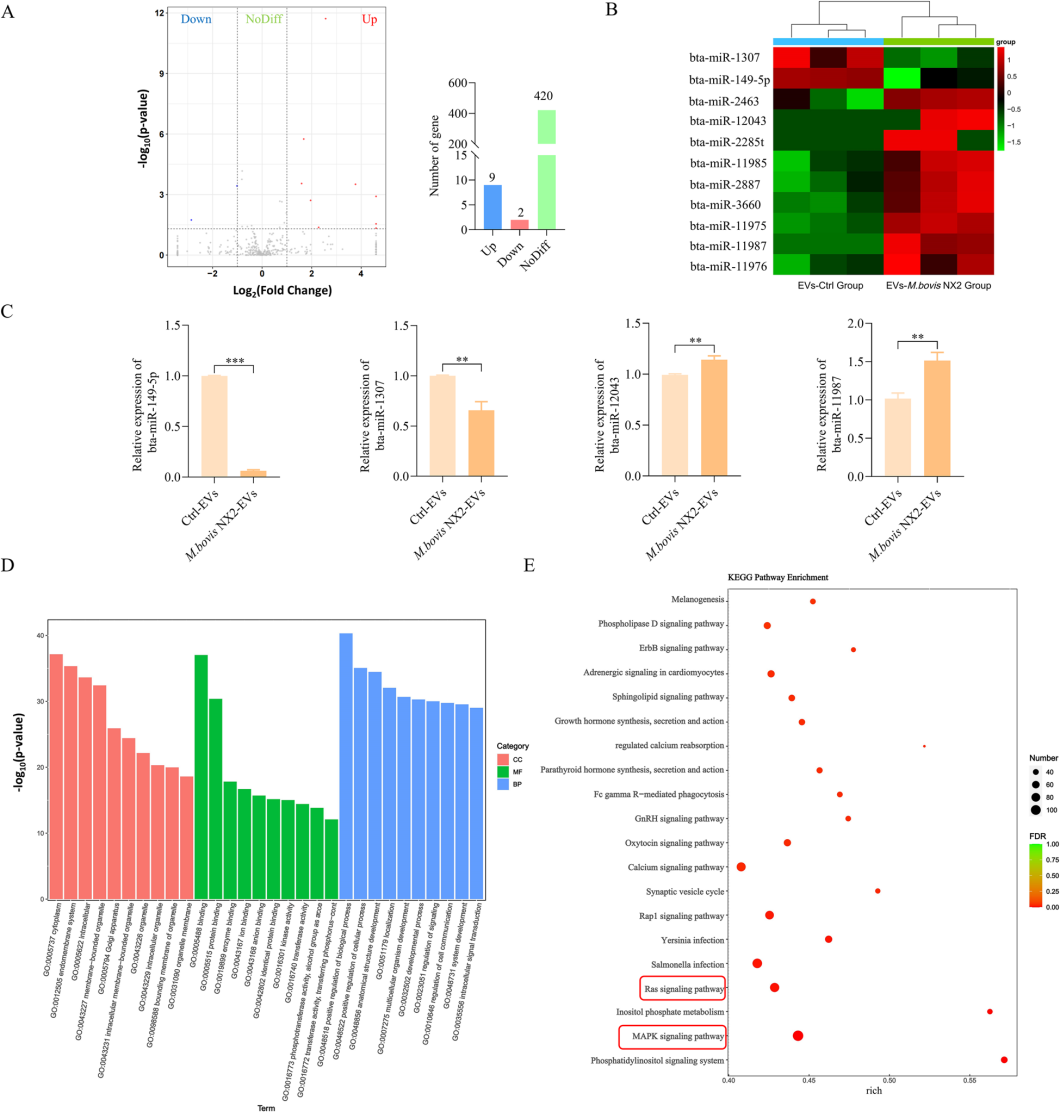
**Analysis and characterization of miRNA expression profiles in Ctrl-EVs and** ***M. bovis*** **NX2-EVs**. **A** Volcano plots and bar graphs showing differentially expressed miRNAs between Ctrl-EVs and *M. bovis* NX2-EVs. Red and blue dots indicate significantly upregulated miRNAs and downregulated miRNAs, respectively, in *M. bovis NX2*-EVs compared with Ctrl-EVs, whereas grey dots indicate miRNAs whose expression did not significantly differ. **B** Heatmap showing differentially expressed miRNAs between Ctrl-EVs and *M. bovis NX2*-EVs, with red and green representing upregulated and downregulated miRNAs, respectively, in *M. bovis NX2*-EVs. **C** The expression of bta-miR-11987, bta-miR-12043, bta-miR-1307 and bta-miR-149-5p was measured by qRT‒PCR. **D** GO enrichment analysis of the predicted target genes of the differentially expressed miRNAs. **E** KEGG enrichment analysis of the predicted target genes of the differentially expressed miRNAs. The results are presented as the mean ± SEM from three independent experiments, and a two-tailed Student’s *t* test used to analyse the data shown in (**C**); **p < 0.01 and ***p < 0.001 indicate a significant difference.

miRNAs are among the major cargoes of EVs and are crucial for cell-to-cell communication. Given that *M. bovis* NX2-EVs can induce an inflammatory response in BoMacs, it was hypothesized that *M. bovis* NX2 infection alters the miRNA composition of EVs. To investigate whether *M. bovis* NX2-EVs harbour specific miRNAs that regulate the inflammatory response, small RNA sequencing was performed before the differentially expressed miRNAs between Ctrl-EVs and *M. bovis* NX2-EVs were analysed. Compared with those in the Ctrl-EVs, a total of nine miRNAs in the *M. bovis* NX2-EVs (bta-miR-2887, bta-miR-11987, bta-miR-11976, bta-miR-11985, bta-miR-2463, bta-miR-11975, bta-miR-12043, bta-miR-3660, and bta-miR-2285t) were upregulated, while 2 miRNAs (bta-miR-1307 and bta-miR-149-5p) were downregulated (Figures 7A and B). To validate the sequencing data, the expression levels of bta-miR-1307, bta-miR-149-5p, bta-miR-11987 and bta-miR-12043 in Ctrl-EVs and *M. bovis* NX2-EVs were measured by qRT‒PCR. Consistent with the miRNA-seq results, bta-miR-11987 and bta-miR-12043 were significantly enriched in *M. bovis* NX2-EVs compared with Ctrl-EVs. Conversely, bta-miR-1307 and bta-miR-149-5p were expressed at significantly lower levels in *M. bovis* NX2-EVs than in Ctrl-EVs (Figure 7C). The 3'UTR' sequence of the miRNAs was used as the target sequence to predict their corresponding target genes. This was followed by GO enrichment analysis to determine genes that were enriched for signalling regulation, regulation of cellular communication, and intracellular signal transduction (Figure 7D). Furthermore, KEGG enrichment analysis indicated that the differentially expressed miRNA target genes were enriched primarily in the MAPK and Ras signalling pathways (Figure 7E). Overall, the results suggest that differentially expressed miRNAs may activate the MAPK and Ras signalling pathways by binding to target genes in BoMacs.

## Discussion

*M. bovis* is a pathogen that poses a significant threat to the global cattle industry worldwide, causing various diseases, such as bovine pneumonia, mastitis and arthritis, that result in substantial economic losses [1, 2]. Epithelial cells act as the first line of defence against pathogen infection. Upon *M. bovis* infection, the organism adheres to and invades mammary gland epithelial cells, prompting the upregulation of proinflammatory chemokines and cytokines by MAC-T cells [26, 28, 32]. Additionally, *M. bovis* activates bovine macrophages, leading to the secretion of proinflammatory cytokines, such as TNF-α, IL-4 and IFN-γ. These cytokines, in turn, induce inflammatory responses that cause pathological immune injuries, thus highlighting their critical role in the pathogenesis of *M. bovis* [12, 33, 34].

EVs, membrane-encapsulated and heterogeneous particles secreted by all cell types, are key mediators of intercellular communication that modulate cellular immune responses by transporting biomolecules, such as nucleic acids, RNAs and proteins, to target cells [35]. EVs interact with and are internalized by target cells through different mechanisms, including endocytosis, phagocytosis and macrocytosis [17–19]. In this study, BoMacs were found to take up MAC-T-derived EVs primarily through endocytosis and macrocytosis. Numerous studies have shown that EVs derived from various cells are crucial for regulating the secretion of inflammatory cytokines from target cells. For example, EVs derived from tumour cells regulate the inflammatory responses of nearby or distal immune cells [36], whereas those obtained from COM-treated macrophages stimulated the release of IL-8 by renal tubular cells and monocytes [37]. Furthermore, treating naïve mice with EVs isolated from the serum of LPS-treated mice increased the levels of the proinflammatory cytokines IL-6 and TNF-α in the lungs of the injected animals [38]. Similarly, in other studies, EVs purified from the semen of fertile males stimulated the release of IL-8 and IL-6 by human endometrial stromal cells [39], whereas those released from LPS-treated RAW264.7 cells increased the expression of caspase-1, ASC and NLRP3 after being taken up by AML-12 hepatocytes. These findings suggest that EVs secreted by cells exposed to external factors may carry immunostimulatory molecules that elicit inflammatory responses in target cells. Consistent with the above data, the current results demonstrated that EVs released from *M. bovis*-infected MAC-T cells could induce the expression of TNF-α, IL-8, IL-6 and IL-1β in BoMacs. It was also noted that the DEGs were mainly enriched in NF-kappa B, TNF and IL-17 signalling pathways in BoMacs following treatment with *M. bovis* NX2-EVs. GSEA subsequently revealed the enrichment of pathways associated with Toll-like receptors and NF-kappa B, TNF and IL-17 signalling in the same cells. Notably, the NF-kappa B pathway is a critical mediator of innate immunity and inflammation, especially because it drives the secretion of inflammatory cytokines to regulate the immune responses of cells. Recently, studies have revealed that intramammary *M. bovis* infection induces the expression of TNF-α, IL-1β, IL-6, and TGF-α in mammary gland tissues and milk [40, 41]. Similarly, we found that EVs derived from *M. bovis* NX2-infected MAC-T cells can increase the expression of TNF-α, IL-1β, and IL-6 in BoMacs, BMDMs, and bovine monocyte-derived macrophages. Therefore, we propose that EVs derived from *M. bovis* NX2-infected MAC-T cells increase the development of mastitis. However, the precise mechanism underlying this response requires further investigation.

Interestingly, several studies have shown that various factors can stimulate the release of EVs [42]. For instance, *Mycobacterium tuberculosis* infection triggers the release of EVs from neutrophils, which subsequently promotes macrophage autophagy [43]. Similarly, *Cryptococcus* induces macrophages to secrete EVs that facilitate BEAS-2B cell death [44]. Consistent with these observations, this study revealed an increased abundance of EVs in MAC-T cells treated with *M. bovis* NX2. However, whether the amount of EVs released locally during infection is sufficient to trigger an inflammatory response remains unclear. More importantly, differences in the composition of EVs produced by pathogen-infected hosts are crucial for modulating the response of immune cells to infection. On the one hand, high levels of pathogenic proteins are often present in EVs released from pathogen-infected cells, and these proteins are subsequently involved in the induction of proinflammatory responses. For instance, EVs from pathogen-infected cells tend to be enriched in proteins, whereas in the case of *M. tuberculosis*-infected THP-1 cells, exosomes containing LAM and *M. bovis* lipoprotein are often released, with these subsequently inducing inflammatory responses in THP-1 cells [45]. Following stimulation by *M. bovis*, J774 cells have also been shown to release exosomes that contain the Ag85 complex, a major secreted protein found in the culture medium of *M. bovis* that is capable of triggering the secretion of the inflammatory cytokines IL-6 and IL-1β [46]. Recently, *M. bovis*-derived EVs elicited the same immune responses in bovine primary blood cells as those induced by live *M. bovis*. In addition, *M. bovis* adhesion to NOX was demonstrated in *M. bovis* EVs [47]. We further detected the presence of NOX in *M. bovis* NX2-EVs to eliminate the contamination of *M. bovis* EVs. The results revealed that the *Mycoplasma* adhesion-associated protein NOX was not detected in *M. bovis* NX2-EVs. These data indicate that *M. bovis* NX2-EVs can induce inflammatory responses in BoMacs, BMDMs, and bovine monocyte-derived macrophages in the absence of *M. bovis*-derived proteins. In contrast, high levels of host vesicle components have also been identified in EVs released from pathogen-infected cells, and these components are often involved in inducing proinflammatory responses. In this context, *Tannerella forsythia*-infected macrophage-derived EVs were found to harbour high levels of inflammatory mediators, which induced the expression of proinflammatory cytokines in THP-1 cells [48]. Similarly, EVs lacking gga-miR-451 and derived from *Mycoplasma gallisepticum* (MG)-infected CP-II cells increase the production of inflammatory cytokines in chicken fibroblasts (DF-1) [24]. Therefore, based on the findings, it was hypothesized that EVs released from *M. bovis*-infected MAC-T cells carry inflammatory mediators, which may contribute to the pathogenesis of bovine mastitis and related diseases.

A comprehensive and comparative analysis of the composition and function of EV cargo proteins and miRNAs from *M. bovis*-infected And uninfected MAC-T cells was performed. Overall, 27 And 86 proteins were significantly downregulated and upregulated, respectively, in *M. bovis* NX2-EVs compared with Ctrl-EVs. These differentially expressed proteins were enriched in signalling pathways, such as the NF-kappa B, IL-17, and TNF pathways. Furthermore, the protein expression of JCHAIN, JAG1, MAPRE1 and ARCN1 was significantly enriched in *M. bovis* NX2-EVs, and immunogold labelling indicated that these proteins were localized on the membrane of EVs. Moreover, JCHAIN was the most enriched protein, with involvement in cellular immune regulation and related diseases. It was previously reported that JCHAIN was involved in breast cancer invasion and metastasis through regulation of the NF-kappa B signalling pathway [49]. Similarly, it was the most abundant protein produced in vivo and was found to be involved in intestinal homeostasis and mucosal immunity [50]. It has been reported that microtubule-associated protein RP/EB family member 1 (MAPRE1) interacts with HIV-1 regulatory viral proteins and impairs the immune function of HIV-1-infected macrophages [51]. Numerous studies have shown that JAG1-mediated Notch signalling is crucial for regulating human airway epithelial cell differentiation, pulmonary fibrosis and other diseases [52, 53]. However, their potential to regulate inflammatory responses during *M. bovis* infection has not been demonstrated. In addition, several studies have indicated that certain membrane proteins, such as Cav-1, nSMase2, and Vps4A, are involved in the biogenesis of EVs [54]. Surprisingly, studies have reported that the JAG1 protein promotes EV secretion and is involved in cargo sorting [55]. Further studies are needed to elucidate the potential roles of these membrane proteins in cargo sorting. Taken together, these results suggest that the most enriched proteins, JCHAIN and MAPRE1, in *M. bovis* NX2-EVs may induce an inflammatory response in BoMacs.

Subsequent miRNA analysis revealed that nine miRNAs were upregulated and two were downregulated in *M. bovis* NX2-EVs compared with Ctrl-EVs, suggesting that the infection influenced the expression of EV miRNAs in MAC-T cells. miRNAs are important for regulating the interactions between pathogens and host cells [56]. Among these differentially expressed miRNAs, bta-miR-1307 and bta-miR-149-5p were have lower expression levels in *M. bovis* NX2-EVs than in Ctrl-EVs. Additionally, bta-miR-11987 and bta-miR-12043 were enriched in *M. bovis* NX2-EVs; notably, in foot-and-mouth disease, miR-1307 attenuates the replication of FMDV by destabilizing the viral structural protein VP3 and enhancing the host innate immune response [57]. Additionally, osteosarcoma (OS) cell-derived EV miR-1307 can promote the proliferation, migration and invasion of OS cells [58], whereas other reports highlight the role of miR-149-5p in the development of metabolic dysfunction-associated steatohepatitis (MASLD) via multiple metabolic and inflammatory pathways in hepatocytes [59, 60]. However, studies on the function of miR-11987 and miR-12043 are limited. Furthermore, KEGG enrichment analysis revealed that the differentially expressed miRNA target genes were enriched primarily in the MAPK and Ras signalling pathways. MAPK is important for inducing, promoting and regulating inflammatory responses in the immune system. These results suggest that the expression of the miRNAs bta-miR-1307 and bta-miR-149-5p could be linked to the modulation of inflammatory responses during *M. bovis* infection. Although this study did not generate evidence in support of this link, the investigation of the potential molecular mechanisms linking bta-miR-1307 and bta-miR-149-5p to the MAPK and Ras pathways in target cells deserves further exploration.

In conclusion, this study revealed that EVs derived from *M. bovis*-infected MAC-T cells promote inflammatory responses in bovine macrophages. Furthermore, the enriched proteins JCHAIN and MAPRE1 as well as the low-abundance miRNAs bta-miR-1307 and bta-miR-149-5p could be involved in regulating the inflammatory response. These results enhance the current knowledge of *M. bovis*–host interactions and reveal the mechanism of host resistance to infection while providing new insights for exploring the pathogenic mechanism of *M. bovis*.

## Supplementary Information

**Additional file 1.** ***Mycoplasma bovis* NX2 survival in DMEM/F-12 and PPLO**. CFU counts taken at different time points. (A) *M. bovis* NX2 was grown in PPLO. (B) *M. bovis* NX2 was grown in DMEM/F-12 (10% FBS). The results are presented as the mean ± SEM from three independent experiments; Student’s *t* test was used to analyse the data. *** *p* < 0.001 indicates a significant difference; ns indicates no difference.

**Additional file 2.* Mycoplasma bovis* NX2-EVs induced TNF-α expression in BoMacs at 38.5 °C**. (A) western blotting was used to measure TNF-α levels in BoMacs incubated with 50 μg/mL Ctrl-EVs and *M. bovis* NX2-EVs at 38.5 °C for 24 h. (B) The intensity of the TNF-α band relative to that of the GAPDH band was analysed by ImageJ software. The results are presented as the mean ± SEM from three independent experiments; Student’s *t* test was used to analyse the data. ** p < 0.01 indicates a significant difference; ns indicates no difference.

## Data Availability

The RNA-seq and miRNA-seq data have been deposited in the Gene Expression Omnibus (GEO) database under the accession codes GSE281774 and GSE281987. The mass spectrometry proteomic data have been deposited in the iProX with the dataset identifier PXD057725. The datasets used and/or analysed during the current study are available from the corresponding author upon reasonable request.

## References

[1] Askar H, Chen S, Hao H, Yan X, Ma L, Liu Y, Chu Y (2021) Immune evasion of *Mycoplasma bovis*. Pathogens 10:297 33806506 10.3390/pathogens10030297 PMC7998117

[2] Suwanruengsri M, Uemura R, Kanda T, Fuke N, Nueangphuet P, Pornthummawat A, Yasuda M, Hirai T, Yamaguchi R (2022) Production of granulomas in *Mycoplasma bovis* infection associated with meningitis-meningoencephalitis, endocarditis, and pneumonia in cattle. J Vet Diagn Invest 34:68–76 34802307 10.1177/10406387211053254 PMC8689037

[3] Cantón G, Llada I, Margineda C, Urtizbiría F, Fanti S, Scioli V, Fiorentino MA, Louge Uriarte E, Morrell E, Sticotti E, Tamiozzo P (2022) *Mycoplasma bovis*-pneumonia and polyarthritis in feedlot calves in Argentina: first local isolation. Rev Argent Microbiol 54:299–304 35606271 10.1016/j.ram.2022.02.005

[4] Gelgie AE, Korsa MG, Kerro Dego O (2022) *Mycoplasma bovis* mastitis. Curr Res Microb Sci 3:100123 35909617 10.1016/j.crmicr.2022.100123 PMC9325741

[5] Dudek K, Nicholas RAJ, Szacawa E, Bednarek D (2020) *Mycoplasma bovis* infections-occurrence, diagnosis and control. Pathogens 9:640 32781697 10.3390/pathogens9080640 PMC7459460

[6] Hale HH, Helmboldt CF, Plastridge WN, Stula EF (1962) Bovine mastitis caused by a *Mycoplasma* species. Cornell Vet 52:582–591 13952069

[7] Zhu X, Baranowski E, Dong Y, Li X, Hao Z, Zhao G, Zhang H, Lu D, Rasheed MA, Chen Y, Hu C, Chen H, Sagné E, Citti C, Guo A (2020) An emerging role for cyclic dinucleotide phosphodiesterase and nanoRNase activities in *Mycoplasma bovis*: Securing survival in cell culture. PLoS Pathog 16:e1008661 32598377 10.1371/journal.ppat.1008661 PMC7373297

[8] Guo M, Wang G, Lv T, Song X, Wang T, Xie G, Cao Y, Zhang N, Cao R (2014) Endometrial inflammation and abnormal expression of extracellular matrix proteins induced by *Mycoplasma bovis* in dairy cows. Theriogenology 81:669–674 24439165 10.1016/j.theriogenology.2013.10.004

[9] Wang Y, Liu S, Li Y, Wang Q, Shao J, Chen Y, Xin J (2016) *Mycoplasma bovis*-derived lipid-associated membrane proteins activate IL-1β production through the NF-κB pathway via toll-like receptor 2 and MyD88. Dev Comp Immunol 55:111–118 26499291 10.1016/j.dci.2015.10.017

[10] Schneider P, Brill R, Schouten I, Nissim-Eliraz E, Lysnyansky I, Shpigel NY (2022) Lipoproteins are potent activators of nuclear factor kappa B in mammary epithelial cells and virulence factors in *Mycoplasma bovis* mastitis. Microorganisms 10:2209 36363800 10.3390/microorganisms10112209 PMC9693531

[11] Hermeyer K, Jacobsen B, Spergser J, Rosengarten R, Hewicker-Trautwein M (2011) Detection of *Mycoplasma bovis* by in-situ hybridization and expression of inducible nitric oxide synthase, nitrotyrosine and manganese superoxide dismutase in the lungs of experimentally-infected calves. J Comp Pathol 145:240–250 21334636 10.1016/j.jcpa.2010.12.005

[12] Schott C, Cai H, Parker L, Bateman KG, Caswell JL (2014) Hydrogen peroxide production and free radical-mediated cell stress in *Mycoplasma bovis* pneumonia. J Comp Pathol 150:127–137 24064048 10.1016/j.jcpa.2013.07.008

[13] Khan LA, Miles RJ, Nicholas RA (2005) Hydrogen peroxide production by *Mycoplasma bovis* and *Mycoplasma agalactiae* and effect of in vitro passage on a *Mycoplasma bovis* strain producing high levels of H_2_O_2_. Vet Res Commun 29:181–188 15736853 10.1023/b:verc.0000047506.04096.06

[14] Perez-Casal J (2020) Pathogenesis and virulence of *Mycoplasma bovis*. Vet Clin North Am Food Anim Pract 36:269–278 32327249 10.1016/j.cvfa.2020.02.002

[15] Mohammadipoor A, Hershfield MR, Linsenbardt HR, Smith J, Mack J, Natesan S, Averitt DL, Stark TR, Sosanya NM (2023) Biological function of extracellular vesicles (EVs): a review of the field. Mol Biol Rep 50:8639–8651 37535245 10.1007/s11033-023-08624-w

[16] Kita S, Shimomura I (2022) Extracellular vesicles as an endocrine mechanism connecting distant cells. Mol Cells 45:771–780 36380729 10.14348/molcells.2022.0110 PMC9676990

[17] Zhang H, Wang L, Li C, Yu Y, Yi Y, Wang J, Chen D (2019) Exosome-induced regulation in inflammatory bowel disease. Front Immunol 10:1464 31316512 10.3389/fimmu.2019.01464 PMC6611439

[18] Anand PK, Anand E, Bleck CK, Anes E, Griffiths G (2010) Exosomal Hsp70 induces a pro-inflammatory response to foreign particles including mycobacteria. PLoS One 5:e10136 20405033 10.1371/journal.pone.0010136 PMC2853569

[19] Bhatnagar S, Schorey JS (2007) Exosomes released from infected macrophages contain *Mycobacterium avium* glycopeptidolipids and are proinflammatory. J Biol Chem 282:25779–28789 17591775 10.1074/jbc.M702277200 PMC3636815

[20] Mitsuhashi S, Feldbrügge L, Csizmadia E, Mitsuhashi M, Robson SC, Moss AC (2016) Luminal extracellular vesicles (EVs) in inflammatory bowel disease (IBD) exhibit proinflammatory effects on epithelial cells and macrophages. Inflamm Bowel Dis 22:1587–1595 27271497 10.1097/MIB.0000000000000840 PMC4911338

[21] Tang TT, Wang B, Wu M, Li ZL, Feng Y, Cao JY, Yin D, Liu H, Tang RN, Crowley SD, Lv LL, Liu BC (2020) Extracellular vesicle-encapsulated IL-10 as novel nanotherapeutics against ischemic AKI. Sci Adv 6:eaaz0748 32851154 10.1126/sciadv.aaz0748 PMC7423360

[22] Wang D, Xue H, Tan J, Liu P, Qiao C, Pang C, Zhang L (2022) Bone marrow mesenchymal stem cells-derived exosomes containing miR-539-5p inhibit pyroptosis through NLRP3/caspase-1 signalling to alleviate inflammatory bowel disease. Inflamm Res 71:833–846 35637388 10.1007/s00011-022-01577-z

[23] Lv LL, Feng Y, Wu M, Wang B, Li ZL, Zhong X, Wu WJ, Chen J, Ni HF, Tang TT, Tang RN, Lan HY, Liu BC (2020) Exosomal miRNA-19b-3p of tubular epithelial cells promotes M1 macrophage activation in kidney injury. Cell Death Differ 27:210–226 31097789 10.1038/s41418-019-0349-y PMC7206053

[24] Zhao Y, Fu Y, Zou M, Sun Y, Yin X, Niu L, Gong Y, Peng X (2020) Analysis of deep sequencing exosome-microRNA expression profile derived from CP-II reveals potential role of gga-miRNA-451 in inflammation. J Cell Mol Med 24:6178–6190 32307881 10.1111/jcmm.15244 PMC7294135

[25] Sun Y, Wang Y, Zhao Y, Zou M, Peng X (2021) Exosomal miR-181a-5p reduce *Mycoplasma gallisepticum* (HS strain) infection in chicken by targeting PPM1B and activating the TLR2-mediated MyD88/NF-κB signaling pathway. Mol Immunol 140:144–157 34715577 10.1016/j.molimm.2021.09.005

[26] Josi C, Bürki S, Stojiljkovic A, Wellnitz O, Stoffel MH, Pilo P (2018) Bovine epithelial *in vitro* infection models for *Mycoplasma bovis*. Front Cell Infect Microbiol 8:329 30280094 10.3389/fcimb.2018.00329 PMC6153342

[27] Ogunnaike M, Wang H, Zempleni J (2021) Bovine mammary alveolar MAC-T cells afford a tool for studies of bovine milk exosomes in drug delivery. Int J Pharm 610:121263 34742829 10.1016/j.ijpharm.2021.121263 PMC8665143

[28] Gondaira S, Higuchi H, Iwano H, Nishi K, Nebu T, Nakajima K, Nagahata H (2018) Innate immune response of bovine mammary epithelial cells to *Mycoplasma bovis*. J Vet Sci 19:79–87 28927255 10.4142/jvs.2018.19.1.79 PMC5799403

[29] Zhao G, Zhang H, Chen X, Zhu X, Guo Y, He C, Anwar Khan F, Chen Y, Hu C, Chen H, Guo A (2017) *Mycoplasma bovis* NADH oxidase functions as both a NADH oxidizing and O_2_ reducing enzyme and an adhesin. Sci Rep 7:44 28246386 10.1038/s41598-017-00121-y PMC5427908

[30] Sun X, Gao S, Chang R, Jia H, Xu Q, Mauck J, Loor JJ, Li X, Xu C (2024) Fatty acids promote M1 polarization of monocyte-derived macrophages in healthy or ketotic dairy cows and a bovine macrophage cell line by impairing mTOR-mediated autophagy. J Dairy Sci 107:7423–7434 38754818 10.3168/jds.2023-24357

[31] Démoulins T, Yimthin T, Lindtke D, Eggerschwiler L, Siegenthaler R, Labroussaa F, Jores J (2024) Temperature impacts the bovine *ex vivo* immune response towards *Mycoplasmopsis bovis*. Vet Res 55:18 38351086 10.1186/s13567-024-01272-3 PMC10863263

[32] Yang J, Liu Y, Lin C, Yan R, Li Z, Chen Q, Zhang H, Xu H, Chen X, Chen Y, Guo A, Hu C (2022) Regularity of toll-like receptors in bovine mammary epithelial cells induced by *Mycoplasma bovis*. Front Vet Sci 9:846700 35464378 10.3389/fvets.2022.846700 PMC9021453

[33] Baquero M, Vulikh K, Wong C, Domony M, Burrows D, Marom D, Perez-Casal J, Cai HY, Caswell JL (2021) Effects of inflammatory stimuli on responses of macrophages to *Mycoplasma bovis* infection. Vet Microbiol 262:109235 34530231 10.1016/j.vetmic.2021.109235

[34] Rodríguez F, Castro P, Poveda JB, Afonso AM, Fernández A (2015) Immunohistochemical labelling of cytokines in calves infected experimentally with *Mycoplasma bovis*. J Comp Pathol 152:243–247 25731984 10.1016/j.jcpa.2015.01.006

[35] Kalluri R, LeBleu VS (2020) The biology, function, and biomedical applications of exosomes. Science 367:eaau6977 32029601 10.1126/science.aau6977 PMC7717626

[36] Marar C, Starich B, Wirtz D (2021) Extracellular vesicles in immunomodulation and tumor progression. Nat Immunol 22:560–570 33753940 10.1038/s41590-021-00899-0 PMC9389600

[37] Singhto N, Thongboonkerd V (2018) Exosomes derived from calcium oxalate-exposed macrophages enhance IL-8 production from renal cells, neutrophil migration and crystal invasion through extracellular matrix. J Proteomics 185:64–76 29953960 10.1016/j.jprot.2018.06.015

[38] Jiang K, Yang J, Guo S, Zhao G, Wu H, Deng G (2019) Peripheral circulating exosome-mediated delivery of miR-155 as a novel mechanism for acute lung inflammation. Mol Ther 27:1758–1771 31405809 10.1016/j.ymthe.2019.07.003 PMC6822235

[39] Paktinat S, Hashemi SM, Ghaffari Novin M, Mohammadi-Yeganeh S, Salehpour S, Karamian A, Nazarian H (2019) Seminal exosomes induce interleukin-6 and interleukin-8 secretion by human endometrial stromal cells. Eur J Obstet Gynecol Reprod Biol 235:71–76 30807994 10.1016/j.ejogrb.2019.02.010

[40] Kauf AC, Rosenbusch RF, Paape MJ, Bannerman DD (2007) Innate immune response to intramammary *Mycoplasma bovis* infection. J Dairy Sci 7:3336–3348 10.3168/jds.2007-0058 17582119

[41] Gelgie AE, Gelalcha BD, Freeman T, Ault-Seay TB, Beever J, Kerro Dego O (2025) Whole transcriptome analysis of *Mycoplasma bovis*-host interactions under *in vitro* and *in vivo* conditions. Vet Microbiol 303:110426 39951862 10.1016/j.vetmic.2025.110426

[42] Zhu J, Liu B, Wang Z, Wang D, Ni H, Zhang L, Wang Y (2019) Exosomes from nicotine-stimulated macrophages accelerate atherosclerosis through miR-21-3p/PTEN-mediated VSMC migration and proliferation. Theranostics 9:6901–6919 31660076 10.7150/thno.37357 PMC6815950

[43] Alvarez-Jiménez VD, Leyva-Paredes K, García-Martínez M, Vázquez-Flores L, García-Paredes VG, Campillo-Navarro M, Romo-Cruz I, Rosales-García VH, Castañeda-Casimiro J, González-Pozos S, Hernández JM, Wong-Baeza C, García-Pérez BE, Ortiz-Navarrete V, Estrada-Parra S, Serafín-López J, Wong-Baeza I, Chacón-Salinas R, Estrada-García I (2018) Extracellular vesicles released from *Mycobacterium tuberculosis*-infected neutrophils promote macrophage autophagy and decrease intracellular mycobacterial survival. Front Immunol 9:272 29520273 10.3389/fimmu.2018.00272 PMC5827556

[44] Li X, Xu J, Lin X, Lin Q, Yu T, Chen L, Chen L, Huang X, Zhang X, Chen G, Xu L (2024) Macrophages-derived exo-miR-4449 induced by Cryptococcus affects HUVEC permeability and promotes pyroptosis in BEAS-2B via the HIC1 pathway. Cytokine 173:156441 37995394 10.1016/j.cyto.2023.156441

[45] Bhatnagar S, Shinagawa K, Castellino FJ, Schorey JS (2007) Exosomes released from macrophages infected with intracellular pathogens stimulate a proinflammatory response in vitro and in vivo. Blood 110:3234–3244 17666571 10.1182/blood-2007-03-079152 PMC2200902

[46] Giri PK, Schorey JS (2008) Exosomes derived from *M. bovis* BCG infected macrophages activate antigen-specific CD4+ and CD8+ T cells *in vitro* and *in vivo*. PLoS One 3:e2461 10.1371/journal.pone.0002461 PMC2413420 18560543

[47] Wagner TM, Torres-Puig S, Yimthin T, Irobalieva RN, Heller M, Kaessmeyer S, Démoulins T, Jores J (2025) Extracellular vesicles of minimalistic mollicutes as mediators of immune modulation and horizontal gene transfer. Commun Biol 8:674 40301684 10.1038/s42003-025-08099-4 PMC12041197

[48] Lim Y, Kim HY, Han D, Choi BK (2023) Proteome and immune responses of extracellular vesicles derived from macrophages infected with the periodontal pathogen *Tannerella forsythia*. J Extracell Vesicles 12:e12381 38014595 10.1002/jev2.12381 PMC10682907

[49] Wang M, Wu Y, Li X, Dai M, Li S (2023) IGJ suppresses breast cancer growth and metastasis by inhibiting EMT via the NF-κB signaling pathway. Int J Oncol 63:105 37539706 10.3892/ijo.2023.5553 PMC10552693

[50] Xiong E, Li Y, Min Q, Cui C, Liu J, Hong R, Lai N, Wang Y, Sun J, Matsumoto R, Takahashi D, Hase K, Shinkura R, Tsubata T, Wang JY (2019) MZB1 promotes the secretion of J-chain-containing dimeric IgA and is critical for the suppression of gut inflammation. Proc Natl Acad Sci U S A 116:13480–13489 31127044 10.1073/pnas.1904204116 PMC6613140

[51] Santos da Silva E, Shanmugapriya S, Malikov V, Gu F, Delaney MK, Naghavi MH (2020) HIV-1 capsids mimic a microtubule regulator to coordinate early stages of infection. EMBO J 39:e104870 32896909 10.15252/embj.2020104870 PMC7560205

[52] Gomi K, Staudt MR, Salit J, Kaner RJ, Heldrich J, Rogalski AM, Arbelaez V, Crystal RG, Walters MS (2016) *JAG1*-mediated notch signaling regulates secretory cell differentiation of the human airway epithelium. Stem Cell Rev Rep 12:454–463 27216293 10.1007/s12015-016-9656-6 PMC4926772

[53] Zhao S, Xiao X, Sun S, Li D, Wang W, Fu Y, Fan F (2018) Microrna-30d/JAG1 axis modulates pulmonary fibrosis through Notch signaling pathway. Pathol Res Pract 214:1315–1323 30029934 10.1016/j.prp.2018.02.014

[54] Fabbiano F, Corsi J, Gurrieri E, Trevisan C, Notarangelo M, D’Agostino VG (2021) RNA packaging into extracellular vesicles: an orchestra of RNA-binding proteins? J Extracell Vesicles 10:e12043 10.1002/jev2.12043 PMC7769857 33391635

[55] Xu M, Shi Y, Liu J, Wu M, Zhang F, He Z, Tang M (2023) *JAG1* affects monocytes-macrophages to reshape the pre-metastatic niche of triple-negative breast cancer through LncRNA *MALAT1* in exosomes. Nan Fang Yi Ke Da Xue Xue Bao 43:1525–1535 37814867 10.12122/j.issn.1673-4254.2023.09.10 PMC10563097

[56] Mishra S, Yadav T, Rani V (2016) Exploring miRNA based approaches in cancer diagnostics and therapeutics. Crit Rev Oncol Hematol 98:12–23 26481951 10.1016/j.critrevonc.2015.10.003

[57] Qi L, Wang K, Chen H, Liu X, Lv J, Hou S, Zhang Y, Sun Y (2019) Host microrna miR-1307 suppresses foot-and-mouth disease virus replication by promoting VP3 degradation and enhancing innate immune response. Virology 535:162–170 31306911 10.1016/j.virol.2019.07.009

[58] Han F, Pu P, Wang C, Ding X, Zhu Z, Xiang W, Wang W (2021) Osteosarcoma cell-derived exosomal miR-1307 promotes tumorgenesis via targeting AGAP1. Biomed Res Int 2021:7358153 33834074 10.1155/2021/7358153 PMC8016573

[59] Correia de Sousa M, Delangre E, Berthou F, El Harane S, Maeder C, Fournier M, Krause KH, Gjorgjieva M, Foti M (2024) Hepatic miR-149-5p upregulation fosters steatosis, inflammation and fibrosis development in mice and in human liver organoids. JHEP Rep 6:101126 39263327 10.1016/j.jhepr.2024.101126 PMC11388170

[60] Law YY, Lee WF, Hsu CJ, Lin YY, Tsai CH, Huang CC, Wu MH, Tang CH, Liu JF (2021) MiR-let-7c-5p and miR-149-5p inhibit proinflammatory cytokine production in osteoarthritis and rheumatoid arthritis synovial fibroblasts. Aging (Albany NY) 13:17227–17236 34198264 10.18632/aging.203201 PMC8312412

