# Supplementary figures and images for: Comprehensive multiomic analysis of extracellular vesicles from Mycoplasma bovis-infected bovine mammary epithelial cells identifies proteins and miRNAs that induce inflammatory responses in macrophages
Source: Vet Res. 2025 Sep 25;56:185. doi: 10.1186/s13567-025-01626-5 (PMC12466063; doi:10.1186/s13567-025-01626-5)

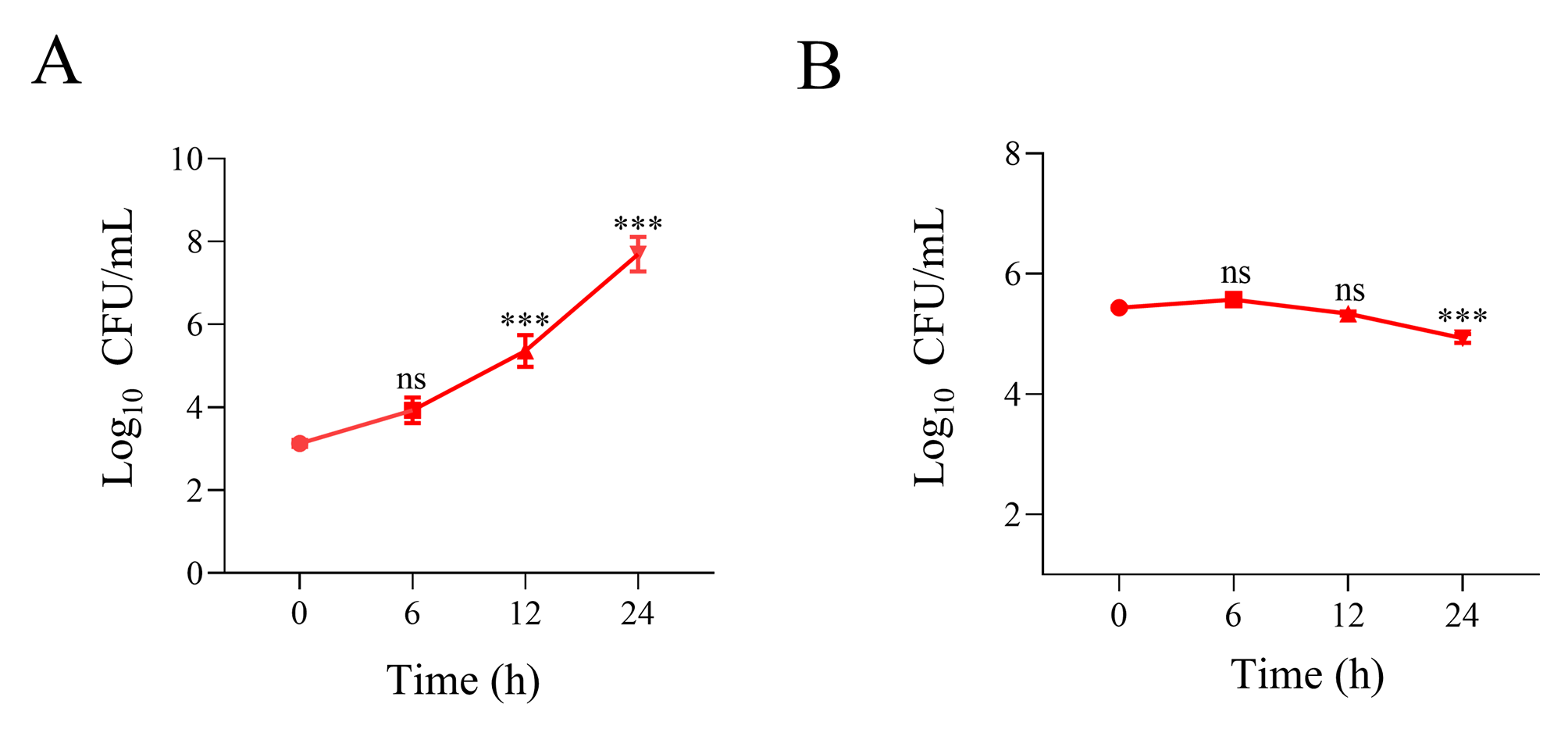

Supplement: Supplementary file 1 — Additional file 1. Mycoplasma bovis NX2 survival in DMEM/F-12 and PPLO. CFU counts taken at different time points. (A) M. bovis NX2 was grown in PPLO. (B) M. bovis NX2 was grown in DMEM/F-12 (10% FBS). The results are presented as the mean ± SEM from three independent experiments; Student’s t test was used to analyse the data. *** p < 0.001 indicates a significant difference; ns indicates no difference. [file 13567_2025_1626_MOESM1_ESM.tif]

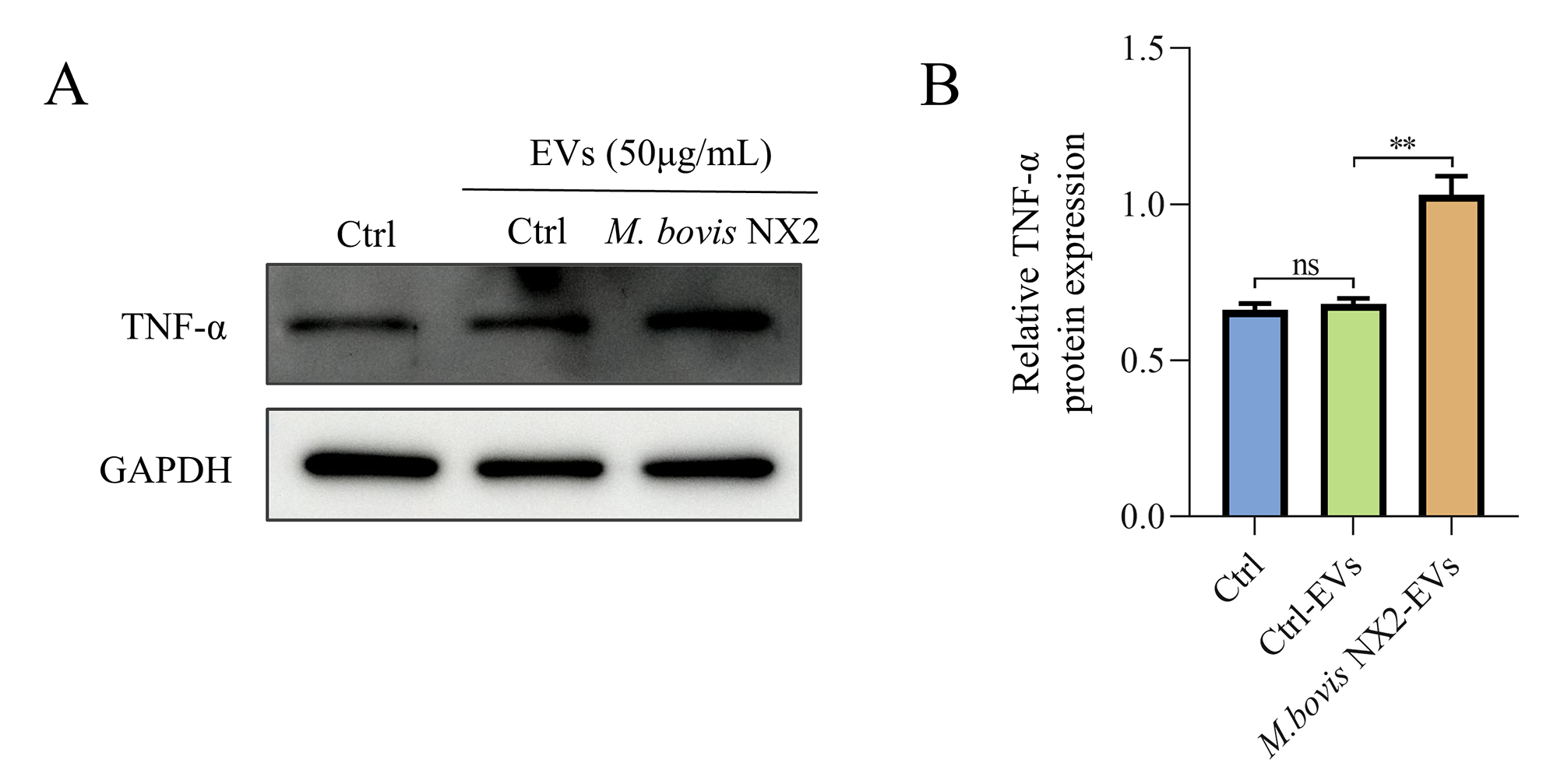

Supplement: Supplementary file 2 — Additional file 2. Mycoplasma bovis NX2-EVs induced TNF-α expression in BoMacs at 38.5 °C. (A) western blotting was used to measure TNF-α levels in BoMacs incubated with 50 μg/mL Ctrl-EVs and M. bovis NX2-EVs at 38.5 °C for 24 h. (B) The intensity of the TNF-α band relative to that of the GAPDH band was analysed by ImageJ software. The results are presented as the mean ± SEM from three independent experiments; Student’s t test was used to analyse the data. ** p < 0.01 indicates a significant difference; ns indicates no difference. [file 13567_2025_1626_MOESM2_ESM.tif]
